# Open-access genomic drug resistance prediction tools for *Mycobacterium tuberculosis*: a systematic review and meta-analysis

**DOI:** 10.1128/jcm.00321-26

**Published:** 2026-07-27

**Authors:** Klaas Dewaele, Christelle Jouego, Adina Asim, Lies Laenen, Conor Meehan, Emmanuel André

**Affiliations:** 1 Unit of Mycobacteriology, Institute of Tropical Medicine 37463 , Antwerp, Belgium; 2 Unit of HIV and Tuberculosis, Institute of Tropical Medicine 37463 , Antwerp, Belgium; 3 Molecular Diagnostic and Research Group, Université de Yaoundé, Yaoundé, Cameroon; 4 Laboratory of Clinical Microbiology, Department of Microbiology, Immunology and Transplantation, KU Leuven 82264, Leuven, Belgium; 5 Department of Biomedical Sciences, Institute of Tropical Medicine 37463 , Antwerp, Belgium; 6 Clinical Department of Laboratory Medicine and National Reference Center for Respiratory Pathogens, KU Leuven University Hospitals Leuven 60182 , Leuven, Belgium; 7 Department of Biosciences, Nottingham Trent University 6122, Nottingham, United Kingdom; University of Western Australia, Perth, Australia

**Keywords:** Mykrobe, MTBseq, TBProfiler, drug resistance, *Mycobacterium tuberculosis*

## Abstract

Whole-genome sequencing (WGS) accelerates drug-susceptibility testing (DST) in *Mycobacterium tuberculosis* (Mtb). Open-access software tools have become widely available, but the sources of real-world performance variability remain uncharacterized. We performed a systematic review and meta-analysis of the performance of open-access, independently validated WGS-based DST prediction tools. Bivariate random-effects meta-analysis was performed for six maintained tools (TBProfiler, Mykrobe, PhyResSE, MTBseq, GenTB, and SAM-TB). Bivariate meta-regression identified covariates associated with performance variation. Thirty-nine studies comprising 144,623 genomes were included. For the two most extensively validated tools, TBProfiler and Mykrobe, pooled rifampicin sensitivity was 95.4% (95% CI: 93.5–96.7) and 93.7% (92.0–95.1), with a specificity of 97.3% (95.7–98.3) and 97.0% (94.8–98.3), respectively. For isoniazid, the sensitivity was 92.0% (90.4–93.3) and 88.2% (85.5–90.4) and specificity 97.3% (96.0–98.2) and 97.5% (95.8–98.5). For ethambutol, the specificity was heterogeneous across tools (86.5%–95.4%); for pyrazinamide, the sensitivity varied widely (49.9%–80.6%). For fluoroquinolones, both sensitivity and specificity approached 90%, with heterogeneity. For newer agents, data scarcity precluded meaningful assessment. Meta-regression identified rifampicin resistance prevalence as the dominant predictor of decreased specificity across first-line drugs (β −1.5 to −3.6 on logit scale, false discovery rate [FDR] q < 0.05), while lineage composition effects were small and confounded. Current open-access WGS prediction tools achieve clinically useful accuracy as rule-out tests for rifampicin, isoniazid, and fluoroquinolone resistance. Predictive performance for second-line drugs is limited by data scarcity. Methodological limitations, including lineage bias, data leakage, and selective sampling, may undermine the tools’ generalizability across diverse global tuberculosis populations.

**IMPORTANCE:**

Tuberculosis remains a leading infectious disease killer worldwide. Whole-genome sequencing (WGS) of *Mycobacterium tuberculosis* offers the potential to rapidly predict drug resistance as a one-stop test, but the accuracy of the software tools used to interpret sequencing results has been inconsistently reported. This meta-analysis leverages the heterogeneity across 39 studies and 144,623 genomes to identify factors that drive inconsistencies in reported performance, providing context-specific guidance for clinical adoption. We show that most tools perform adequately as rule-out tests for resistance to the most important first- and second-line drugs but fall short of specificity targets. Importantly, we identify that the local burden of drug resistance in a study population is the dominant factor driving inconsistencies between reported performance estimates. These findings provide guidance for laboratories considering adopting sequencing-based resistance testing and specify priorities for future tool development and validation.

## INTRODUCTION

*Mycobacterium tuberculosis* (Mtb) poses an unrelenting global public health problem. The achieved decline in incidence is projected to fall well short of the World Health Organization (WHO)’s target of a 50% reduction by 2025, presently reaching only an 8.3% reduction compared to 2015 (1). Early diagnosis, accurate drug susceptibility testing (DST), and contact tracing are among the key components of WHO’s End TB Strategy (2). Despite its long-standing status as a major global public health problem, progress in the field of Mtb diagnosis and DST has been limited over the past half century. Phenotype-based methods, which rely on crude measures of bacterial growth or metabolism, were first developed in the 1960s and remain the reference standard in susceptibility testing for Mtb (3). These rely on Mtb’s slow *in vitro* growth, are technically demanding, and, for some drugs (e.g., ethambutol and pyrazinamide), exhibit poor reproducibility (4). Since the late 2000s, molecular methods have improved the accessibility and turnaround time of drug susceptibility testing in Mtb. Among these, whole-genome sequencing (WGS) provides the most comprehensive diagnostic picture, enabling phylogenetic classification, transmission cluster analysis, and drug susceptibility prediction (5, 6). Compared to rapid genotypic tests or targeted sequencing, WGS enables genome-wide detection of resistance-associated variants, improving sensitivity (7). Compared to phenotypic DST, a WGS-based workflow offers a reduction of time-to-result from months down to days (8, 9). A multicenter study demonstrated the cost-effectiveness of a WGS-based diagnostic workflow for a large proportion of the strains encountered in the clinic, offering a median time gain of 21 days compared to routine diagnosis, and 7% annual cost savings (8). As a result, several national public health institutions have transitioned from phenotypic to primarily WGS-based diagnostics (10–12).

Amid these advances, the role of WGS-based diagnosis in lower tiers of the tuberculosis healthcare systems has yet to be established. While sequencing technology has become significantly more affordable and portable, deriving clinically usable results from sequencing data remains technically demanding. In well-resourced reference laboratories, this process typically involves custom-built bioinformatics pipelines. Such expertise is often unavailable in smaller laboratories or resource-constrained settings. To address this need, freely accessible, sample-to-answer bioinformatic pipelines have been developed. Early versions were experimental and differed widely in diagnostic scope, performance, and ease of use (13). In recent years, several of these tools have matured into integrated applications, some providing identification, high-resolution typing, and drug susceptibility prediction from sequencing data, while requiring minimal or no bioinformatics expertise or infrastructure (6, 14). Concurrent with the advent of affordable and portable sequencing platforms, these tools hold the potential for enabling genomics-informed Mtb diagnosis in resource-limited settings, where tuberculosis burden is often highest. Nevertheless, performance data have varied significantly between studies, some reporting near-perfect sensitivity and specificity, particularly for first-line drugs like rifampicin and isoniazid (6, 13, 15), while others report below-target figures (14, 16). While previous meta-analyses have pooled performance data for a subset of tools (15, 17) and small benchmarking studies have compared pipelines head-to-head on harmonized data sets (18), the independent drivers of cross-study performance variability remain uncharacterized. This work aimed to conduct a meta-analysis of the performance of open-access genomic prediction tools for TB DST and dissect sources of between-study heterogeneity.

## MATERIALS AND METHODS

This study was registered in the PROSPERO database with identifier CRD42023430709.

### Eligibility criteria

We conducted a systematic review and meta-analysis of the performance of open-access software tools for genomic drug susceptibility prediction in *M. tuberculosis*. Tools were considered for inclusion if they met the following criteria: their use is free of charge and open to the public (with or without user registration). Tools may be web-based or run on a local system. They rely on raw genomic data (FASTQ or FAST5 format) and must deliver clinically interpretable susceptibility predictions. They must be tailored for susceptibility prediction in Mtb.

### Information sources

We queried Medline, Embase, and Web of Science on the 29th of October 2025, without language restrictions.

### Search strategy

Our search strategy involved, first, the identification of a core set of eight often-used tools meeting our criteria. Through citation-chaining of key publications for these tools, we then identified 14 newer or lesser-known tools. To be included, we required a tool to have been validated at least once by an independent group. Using this criterion, we compiled a list of twelve eligible tools: KvarQ (19), TGS-TB (20), CASTB (21), PhyResSE (22), ReSeqWHO and its predecessor ReSeqTB (23), MTBseq (24), Mykrobe (6) and its predecessor, Mykrobe Predictor (25), TBProfiler (5, 26), GenTB (14), MAGMA (27), PointFinder (28), and SAM-TB (29). A composite search term was used to identify publications that contained any of the names of these tools in the title or abstract. The details of this procedure and the search term are described in File S1.

### Selection process

**Fig 1:**
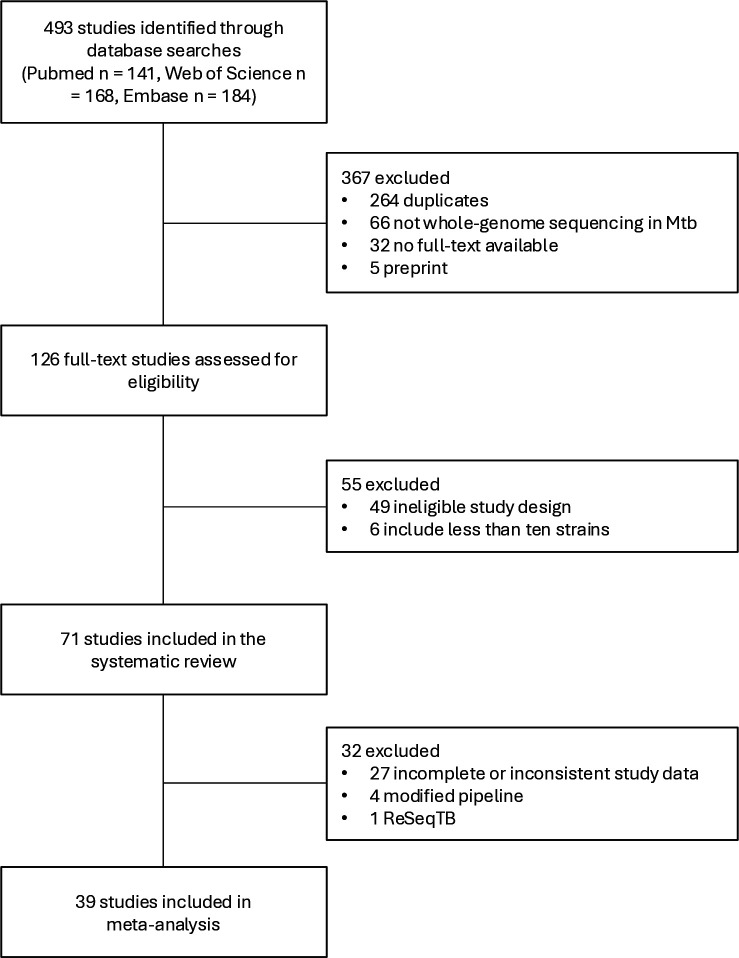
Study selection.

Records were screened by two blinded reviewers (KD and CJ). The selection process is shown in Fig. 1. Only publications that compared performance with phenotypic drug susceptibility testing as a standard and that studied at least ten strains were retained. Both Illumina- (Illumina, CA, USA) and Oxford Nanopore-sequenced (Oxford Nanopore, Oxford, UK) strains were eligible for inclusion. We only included studies that evaluated tools using their default settings and in an unmodified state. Congress abstracts were not included due to their lack of methodological details needed for assessing eligibility and study quality.

### Data items

For each tool and tuberculostatic agent, standard diagnostic accuracy outcomes (true/false positives and true/false negatives) were extracted based on predictions of resistance versus susceptibility relative to phenotypic resistance. When not reported, these variables were calculated or approximated using reported sensitivity and specificity data (details given in Table S1). Data were collected for first-line tuberculostatics (isoniazid, rifampicin, ethambutol, and pyrazinamide), for second-line injectables (amikacin, kanamycin, and capreomycin) and fluoroquinolones (ofloxacin, ciprofloxacin, levofloxacin, and moxifloxacin), and when available for other WHO group A, B, and C agents (streptomycin, bedaquiline, cycloserine, para-aminosalicylic acid, rifabutin, ethionamide, prothionamide, and linezolid). Data extraction was performed independently by two blinded reviewers (CJ and KD). Discordances were resolved through deliberation.

### Study risk of bias assessment

The risk of bias and the applicability of studies were appraised using the QUADAS-2 tool (30). The process of appraisal of study quality and details on data extraction are given in File S2 and Tables S2 to S4. Appraisal of bias was done by two independent reviewers (CJ and KD). Discordances were resolved through deliberation.

### Effect measures

We evaluated the diagnostic performance of WGS tools using sensitivity and specificity as the primary effect measures. For each pooled estimate, we calculated 95% confidence intervals (CIs) and represented between-study heterogeneity using the τ^2^ statistic.

### Synthesis methods

We performed a bivariate random-effects meta-analysis to jointly model sensitivity and specificity, accounting for the trade-off and correlation between them, according to recommendations of the Cochrane Diagnostic Test Accuracy Working Group (31). All analyses were conducted in R using the metafor package. As a primary model, we fitted a generalized linear mixed model (GLMM) assuming a binomial distribution for the true positives and false positives (exact likelihood method). We tested two covariance structures for the random effects: unstructured (UN) and heteroscedastic compound symmetry (HCS). The optimal structure was selected based on the Akaike Information Criterion (AIC), favoring parsimonious models (HCS) when AIC differences were negligible (<2). As a fallback model, in cases where the GLMM failed to converge, produced singular variances, or estimated correlations near the boundary (|ρ| >0.995), we used an Approximate Normal (Two-Stage) model (rma.mv) using the Sweeting correction (adding 0.5 to cells only in studies with zero counts) to stabilize variance estimates. Finally, for combinations with insufficient studies for variance estimation (*N* < 3) or where both meta-analytical models failed to converge, we calculated simple pooled estimates (summing raw counts across studies) with 95% Wilson Score intervals. These pooled estimates do not account for between-study heterogeneity. Data points for a specific tool-drug combination were included in the synthesis only if they were based on at least five isolates.

### Exploration of heterogeneity

We investigated potential sources of statistical heterogeneity (τ^2^) using bivariate meta-regression for the following covariates: publication year, sample size, data source (original vs public), rifampicin resistance prevalence, geographic origin, lineage distribution, phenotypic DST method (solid vs liquid), and tool version. To maximize statistical power, we used multilevel random-effects models with nested random effects (Study/Tool) where studies evaluated multiple tools. We assessed significance using Z-tests for continuous variables and Omnibus tests (QM) for categorical variables.

To address potential confounding, we performed a change-in-estimate analysis, comparing unadjusted β coefficients with those adjusted for rifampicin resistance prevalence. Finally, we identified independent predictors of specificity using multivariate meta-regression for each first-line drug. These models adjusted for resistance prevalence, data source, geographic region, Lineage 2 proportion, pDST method, and publication year. Due to data sparsity in multivariate combinations, these final models were stratified by drug and pooled across tools using standard random-effects models without nested effects.

### Systematic review

Per tool, we systematically extracted the tool’s characteristics regarding preprocessing, genome assembly, variant calling, and variant annotation, as well as any aspects related to clinical utility (Table S5 and File S3).

### Role of the funding source

There was no funding source for this study.

## RESULTS

### Meta-analysis

#### Characteristics of included studies and tools

One hundred and twenty-six articles were assessed for eligibility, of which 39 were included in the meta-analysis (Fig. 1). An overview of the included studies is shown in Table 1.

**TABLE 1:** Characteristics of the included studies^*a*^

Study ID	Region	Source	Number of isolates	Drugs evaluated	Tools evaluated	RIF res %	L1 %	L2 %	L3 %	L4 %
Bradley 2015 (25)	Global	Mixed	1,586	H, R, E, and S; Am, Cfx, Cm, Km, and Mfx	KvarQ and Mykrobe	10.8	NR	NR	NR	NR
Chatterjee 2017 (32)	South Asia	Original	29	H, R, Z, and E	Mykrobe	37.9	6.8	36.5	33.8	23
Che 2022 (33)	Southeast Asia	Original	59	H, R, E, and S; Am, Cm, and Lfx; Pas and Pto	TB-Profiler	42.7	0	89.8	0	10.2
Chen 2025 (17)	Global	Public	33,224	H, R, Z, and E; Am, Cm, Km, and Lfx; Eto	GenTB-RF, SAM-TB, and TB-Profiler	18.6	9.4	26.6	14.9	47.1
Cloutier Charette 2024 (34)	Africa	Original	72	H, R, Z, E, and S; Am, Cm, FQ, and Km; Eto and Lzd	Mykrobe	26.7	NR	NR	NR	NR
Coll 2015 (26)	Global	Original	694	H, R, Z, E, and S; Am, Cm, Km, Mfx, and Ofx; Eto	TB-Profiler	36.7	8.6	23	10.9	57.6
Daniyarov 2023 (35)	Central Asia	Original	10	H, R, Z, E, and S; Am, Cfx, Cm, FQ, Km, Lfx, Mfx, and Ofx; Eto and Pas	CASTB, Mykrobe, ResFinder, and TB-Profiler	50.4	0	100	0	0
Deelder 2022 (36)	Global	Public	2,118	H, R, and E; Cs, Eto, and Pas	TB-Profiler	14.5	9.6	25.2	11.4	51
Gröschel 2021 (14)	Global	Public	19,542	H, R, Z, E, and S; Am, Cfx, Cm, Km, Lfx, and Ofx; Eto and Pas	GenTB-RF, GenTB-WDNN, Mykrobe, and TB-Profiler	21.5	10	21	15	52
Guimaraes 2020 (37)	South America	Mixed	71	H, R, Z, E, and S	CASTB, KvarQ, MTBseq, Mykrobe, PhyResSE, and TB-Profiler	19.6	100	0	0	0
Hall & Lima 2023 (38)	Global	Public	40,548	H, R, Z, E, and S; Am, Cm, Km, Lfx, Mfx, and Ofx; Dlm, Eto, and Lzd	Mykrobe	16.5	8.7	28.8	13	46.4
Hall & Rabodoarivelo 2023 (38)	Africa	Original	99	H, R, E, and S; Am, Cm, Km, and Ofx	Mykrobe	19.2	NR	NR	NR	NR
Hasan 2023 (39)	Not reported	Public	1,526	H, R, Z, and E	TB-Profiler	0	NR	NR	NR	NR
He 2024 (40)	Southeast Asia	Public	110	H, R, Z, E, and S; Am, Cm, and Km; Pas and Pto	GenTB-RF, Mykrobe, PhyResSE, SAM-TB, and TB-Profiler	46	0	79.1	0	20.9
Hunt 2019 (6)	Europe	Original	4,308	H, R, Z, E, and S; Am, Cfx, Cm, FQ, Km, Mfx, and Ofx; Cs, Lzd, Pas, and Rfb	KvarQ, MTBseq, Mykrobe, and TB-Profiler	15.2	NR	NR	NR	NR
Iwamoto 2019 (41)	Southeast Asia	Original	191	Z	CASTB, PhyResSE, TB-Profiler, and TGS-TB	56.5	NR	NR	NR	NR
Johnsen 2019 (28)	Not reported	Public	2,501	H, R, Z, E, and S; Am, Cm, FQ, and Km; Eto	ResFinder	38.1	NR	NR	NR	NR
Kardan-Yamchi 2020 (42)	West Asia	Original	35	H, R, and E; Am, Cm, Km, Lfx, and Mfx; Bdq, Cfz, Cs, Dlm, Lzd, and Pto	MTBseq	33.7	2.9	34.3	20	42.9
Lim 2023 (43)	Southeast Asia	Original	2,998	H, R, Z, and E	TB-Profiler	3	35	48.5	0	14.1
Liu 2024 (44)	Southeast Asia	Original	297	H, R, Z, E, and S; Am, Cm, FQ, and Km; Eto and Pas	TB-Profiler	35.1	9.8	63.3	0	27
Mahe 2019 (16)	Global	Public	6,464	H, R, Z, E, and S; Am, Cm, FQ, and Km; Eto	Mykrobe and TB-Profiler	25.1	9.5	15	16	59
Morey-Léon 2023(45)	South America	Original	88	H, R, Z, E, and S; Am, Cm, Km, Lfx, and Mfx	KvarQ, Mykrobe, PhyResSE, SAM-TB, and TB-Profiler	34.7	0	0	0	100
Peker 2021 (46)	Europe	Original	15	H, R, Z, E, and S; Am, Cm, FQ, and Km; Eto and Pas	Mykrobe and TB-Profiler	45.9	0	83.3	0	16.7
Phelan 2016 (47)	Europe	Original	11	H, R, Z, E, and S; Am, Cm, Km, Mfx, and Ofx; Eto, Lzd, Pas, and Rfb	Mykrobe and TB-Profiler	69.5	0	0	0	100
Phelan 2019 (5)	Global	Public	17,037	H, R, Z, E, and S; Am, Cfx, Cm, FQ, Km, Mfx, and Ofx; Cs, Eto, and Pas	Mykrobe and TB-Profiler	22.8	10.9	21.6	16.7	49.5
Qu 2024 (48)	Southeast Asia	Original	82	H, R, E, and S; Am, Km, Lfx, and Mfx; Eto and Rfb	Mykrobe	32.7	NR	NR	NR	NR
Quagliaro 2022 (49)	Europe	Original	227	H, R, Z, and E	Mykrobe and PhyResSE	4	13.7	5.3	5.7	68.4
Rukmana 2024 (50)	Southeast Asia	Original	34	Z	GenTB-RF and Mykrobe	23.9	5.9	61.8	0	32.4
Schleusener 2017 (13)	Africa	Public	91	H, R, Z, E, and S	CASTB, KvarQ, Mykrobe, PhyResSE, and TB-Profiler	23.2	4.3	4.3	0	69.6
Sharma 2024 (51)	North America	Original	1,510	H, R, Z, and E	Mykrobe	4.9	NR	NR	NR	NR
Silcocks 2023 (52)	Southeast Asia	Public	1,786	H, R, E, and S	TB-Profiler	18.1	24.8	61.7	0.1	10.5
Vallejos-Sanchez 2025 (53)	South America	Public	2,917	R	MTBseq and TB-Profiler	21.1	NR	NR	NR	NR
Van Beek 2019 (54)	Europe	Original	211	H, R, Z, E, and S	KvarQ, Mykrobe, PhyResSE, TB-Profiler, and TGS-TB	4	NR	NR	NR	NR
Wang & Dong 2025 (55)	Southeast Asia	Original	209	H, R, Z, and E; Am, Cm, Km, and Mfx; Bdq, Cfz, Cs, Dlm, Lzd, Pas, and Pto	TB-Profiler	24.8	2.9	83.2	0	13.9
Wang & Lai 2025 (56)	Southeast Asia	Original	113	H, R, E, and S	Mykrobe, PhyResSE, SAM-TB, and TB-Profiler	28.3	NR	NR	NR	NR
Wang & Yu 2023 (57)	Southeast Asia	Original	202	H, R, E, and S; Cm, Km, and Ofx; Eto	TB-Profiler	47.7	3	83.7	0	13.4
Wu 2020 (58)	Southeast Asia	Original	306	H, R, E, and S; Am and Ofx	TB-Profiler	55.8	0	95.1	0	4.9
Wu 2022 (59)	Southeast Asia	Original	183	H, R, E, and S; Am, Km, Mfx, and Ofx; Cs, Eto, and Pas	TB-Profiler	18.4	0	90.1	0	7.1
Yang 2022 (29)	Not reported	Public	3,119	H, R, Z, E, and S; Am, Cm, Km, Mfx, and Ofx; Cs, Eto, and Pas	SAM-TB and TB-Profiler	44.2	NR	NR	NR	NR

^*a*^ H, isoniazid; R, rifampicin; Z, pyrazinamide; E, ethambutol; S, streptomycin; Am, amikacin; Bdq, bedaquiline; Cm, capreomycin; Cfx, ciprofloxacin; Cfz, clofazimine; Cs, cycloserine; Dlm, delamanid; Eto, ethionamide; FQ, fluoroquinolones; Gfx, gatifloxacin; Km, kanamycin; Lfx, levofloxacin; Lzd, linezolid; Mfx, moxifloxacin; Ofx, ofloxacin; Pas, para-aminosalicylic acid; Pto, prothionamide; Rfb, rifabutin; RIF res,rifampicin resistance. N isolates represents the maximal number of unique strains included in that study; this number may be smaller for individual tuberculostatics; NR = Not reported.

**Fig 2:**
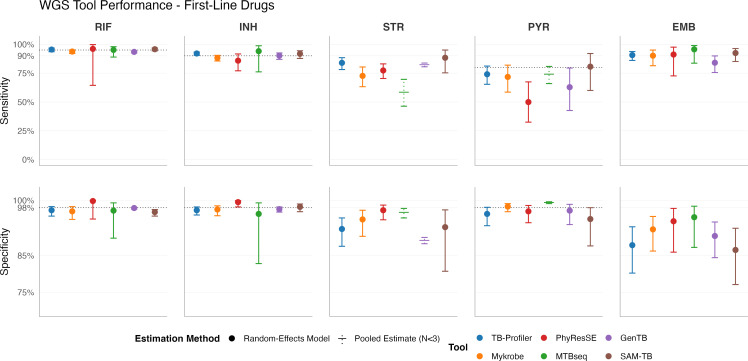
Diagnostic performance of whole-genome sequencing tools for detecting resistance to first-line anti-tuberculosis drugs and streptomycin. Pooled estimates of sensitivity (top panels) and specificity (bottom panels) are shown for each tool across different drugs. Points represent the pooled estimate with 95% confidence intervals. Dotted lines indicate WHO 2021 TPP minimal targets: rifampicin ≥95%/≥98%, isoniazid ≥90%/≥98%, and pyrazinamide ≥80%/≥98% (sensitivity/specificity); no target is defined for streptomycin or ethambutol (62). Dashes denote estimates calculated by pooling raw counts (due to insufficient studies <3 for meta-analysis); these values should be interpreted with caution as they do not account for between-study heterogeneity. INH, isoniazid; RIF, rifampicin; STR, streptomycin; EMB, ethambutol; PYR, pyrazinamide.

**Fig 3:**
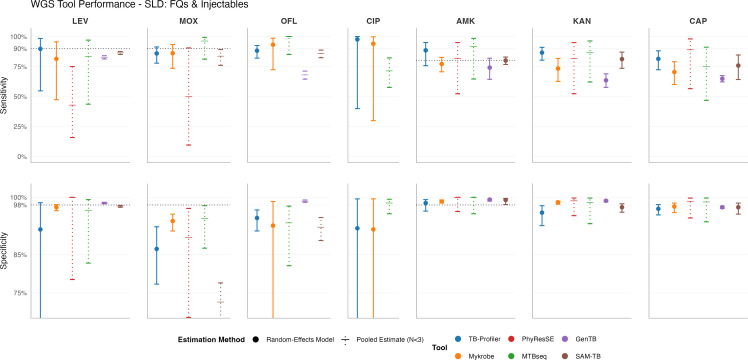
Diagnostic performance of whole-genome sequencing tools for detecting resistance to fluoroquinolones and injectables. Pooled estimates of sensitivity (top panels) and specificity (bottom panels) are shown for each tool across different drugs. Points represent the pooled estimate with 95% confidence intervals. Dotted horizontal lines indicate the 2021 WHO target product profile minimum thresholds for fluoroquinolones (levofloxacin and moxifloxacin; ≥90%/≥98%) and amikacin (≥80%/≥98%); no target has been specified for ofloxacin, ciprofloxacin, kanamycin, or capreomycin (62). Dashes denote estimates calculated by pooling raw counts (due to insufficient studies <3 for meta-analysis); these values should be interpreted with caution as they do not account for between-study heterogeneity. AMK, amikacin; CAP, capreomycin; KAN, kanamycin; CIP, ciprofloxacin; LEV, levofloxacin; MOX, moxifloxacin; OFL, ofloxacin.

**Fig 4:**
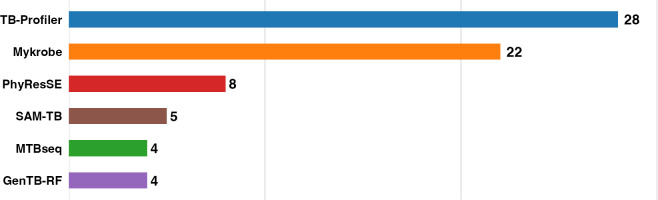
Number of unique validation studies as per the WGS tool. GenTB-RF: publications for the default random forest (RF) model in GenTB.

For MAGMA, no valid evaluation studies were found due to key studies lacking phenotypic comparison (27) or per-drug result reporting (51). For ReSeqTB/ReSeqWHO, no independent validation studies that included more than ten strains were found (23, 60). Additionally, several tools were unavailable or unmaintained in 5 years before the search date (as detailed in Table S5), including CASTB (last update Sep 2017 and inaccessible as of February 2026), TGS-TB (inaccessible since August 2020), and KvarQ (GitHub repository last updated in 2014 [61]). For PointFinder/ResFinder, only two eligible validation studies were available, precluding a robust assessment using a random-effects model. Therefore, the pooled performance was assessed for the following maintained tools: TBProfiler, Mykrobe, PhyResSE, MTBseq, GenTB, and SAM-TB (Fig. 2, first-line tuberculostatics and streptomycin; Fig. 3, fluoroquinolones and second-line injectables; File S1 for other drugs, Table 2; Table S6). Available validation data per tool vary widely; TBProfiler and Mykrobe stand out as being the tools that have been most extensively evaluated (Fig. 4).

**TABLE 2:** Overview of pooled sensitivity and specificity of WGS tools^*a*^,^*b*^

Tool	INH	RIF	EMB	PYR	STR		
GenTB-RF	Se: 90.1 (86.9–92.5) [t²:0.04] Sp: 97.6 (96.8–98.2) [t²:0.04]	Se: 93.4 (92.4–94.3) [t²:0.01] Sp: 97.9 (97.6–98.1) [t²:0.01]	Se: 84.0 (75.5–89.9) [t²:0.18] Sp: 90.3 (84.4–94.1) [t²:0.18]	Se: 62.8 (42.6–79.4) [t²:0.58] Sp: 97.2 (93.4–98.9) [t²:0.58]	* Se: 82.2 (80.5–83.7) [t²:-] Sp: 89.1 (88.2–89.9) [t²:-]		
MTBseq	Se: 94.0 (76.1–98.7) [t²:1.54] Sp: 96.3 (82.8–99.3) [t²:1.54]	Se: 95.1 (88.9–97.9) [t²:0.61] Sp: 97.2 (89.7–99.3) [t²:1.62]	Se: 95.6 (83.6–98.9) [t²:0.64] Sp: 95.4 (87.2–98.4) [t²:0.64]	* Se: 74.0 (65.9–80.8) [t²:-] Sp: 99.4 (99.1–99.6) [t²:-]	* Se: 58.5 (46.3–69.6) [t²:-] Sp: 96.7 (95.2–97.8) [t²:-]		
Mykrobe	Se: 88.2 (85.5–90.4) [t²:0.16] Sp: 97.5 (95.8–98.5) [t²:0.97]	Se: 93.7 (92.0–95.1) [t²:0.12] Sp: 97.0 (94.8–98.3) [t²:1.27]	Se: 90.1 (81.4–95.0) [t²:1.87] Sp: 92.1 (86.2–95.6) [t²:1.87]	Se: 71.7 (58.5–81.9) [t²:0.99] Sp: 98.3 (96.9–99.1) [t²:0.99]	Se: 72.6 (63.2–80.3) [t²:0.64] Sp: 94.8 (90.2–97.3) [t²:1.52]		
PhyResSE	Se: 85.8 (77.0–91.6) [t²:0.39] Sp: 99.5 (98.2–99.9) [t²:0.39]	Se: 96.0 (64.4–99.7) [t²:8.20] Sp: 99.8 (94.9–100.0) [t²:8.20]	Se: 91.1 (72.6–97.5) [t²:1.51] Sp: 94.3 (85.9–97.8) [t²:1.51]	Se: 49.9 (32.5–67.4) [t²:0.62] Sp: 97.0 (93.9–98.6) [t²:0.62]	Se: 77.3 (70.3–83.0) [t²:0.00] Sp: 97.3 (94.7–98.7) [t²:0.00]		
SAM-TB	Se: 91.7 (87.7–94.4) [t²:0.17] Sp: 98.2 (96.9–99.0) [t²:0.17]	Se: 95.7 (94.3–96.8) [t²:0.04] Sp: 96.8 (95.7–97.5) [t²:0.04]	Se: 92.4 (85.1–96.3) [t²:0.46] Sp: 86.5 (77.1–92.4) [t²:0.46]	Se: 80.6 (60.0–92.0) [t²:0.87] Sp: 94.9 (87.6–98.0) [t²:0.87]	Se: 88.3 (75.2–95.0) [t²:0.64] Sp: 92.7 (80.7–97.4) [t²:0.64]		
TB-Profiler	Se: 92.0 (90.4–93.3) [t²:0.14] Sp: 97.3 (96.0–98.2) [t²:0.71]	Se: 95.4 (93.5–96.7) [t²:0.53] Sp: 97.3 (95.7–98.3) [t²:0.96]	Se: 90.6 (86.0–93.7) [t²:0.98] Sp: 87.8 (80.2–92.8) [t²:2.08]	Se: 74.0 (65.3–81.1) [t²:0.65] Sp: 96.3 (93.1–98.1) [t²:1.92]	Se: 83.9 (78.1–88.4) [t²:0.58] Sp: 92.2 (87.5–95.2) [t²:1.05]		

Tool	CIP	OFL	LEV	MOX	AMK	KAN	CAP
GenTB-RF	–	* Se: 68.0 (64.5–71.2) [t²:-] Sp: 99.0 (98.7–99.3) [t²:-]	* Se: 82.7 (81.3–84.0) [t²:-] Sp: 98.5 (98.3–98.7) [t²:-]	–	Se: 74.1 (64.3–81.9) [t²:0.12] Sp: 99.4 (99.0–99.6) [t²:0.12]	Se: 63.5 (57.6–68.9) [t²:0.03] Sp: 99.1 (98.8–99.3) [t²:0.03]	Se: 64.8 (62.2–67.4) [t²:0.00] Sp: 97.4 (97.1–97.7) [t²:0.00]
MTBseq	* Se: 71.4 (57.6–82.2) [t²:-] Sp: 98.5 (95.7–99.5) [t²:-]	* Se: 100.0 (85.1–100.0) [t²:-] Sp: 93.3 (82.1–97.7) [t²:-]	* Se: 83.3 (43.6–97.0) [t²:-] Sp: 96.6 (82.8–99.4) [t²:-]	* Se: 96.2 (81.1–99.3) [t²:-] Sp: 94.5 (86.7–97.8) [t²:-]	* Se: 91.7 (64.6–98.5) [t²:-] Sp: 100.0 (95.7–100.0) [t²:-]	* Se: 86.7 (62.1–96.3) [t²:-] Sp: 98.7 (93.1–99.8) [t²:-]	* Se: 75.0 (46.8–91.1) [t²:-] Sp: 98.8 (93.6–99.8) [t²:-]
Mykrobe	Se: 94.0 (29.9–99.8) [t²:12.69] Sp: 91.6 (33.1–99.6) [t²:9.36]	Se: 93.2 (72.3–98.6) [t²:3.63] Sp: 92.6 (63.8–98.9) [t²:5.42]	Se: 81.4 (47.3–95.6) [t²:1.62] Sp: 97.4 (96.5–98.1) [t²:0.06]	Se: 86.1 (73.5–93.3) [t²:0.63] Sp: 93.8 (91.2-95.6) [t²:0.13]	Se: 77.1 (70.6–82.6) [t²:0.17] Sp: 98.9 (98.5–99.3) [t²:0.17]	Se: 73.3 (62.6–81.8) [t²:0.42] Sp: 98.7 (98.2–99.0) [t²:0.11]	Se: 70.4 (60.0–79.0) [t²:0.34] Sp: 97.6 (96.0–98.5) [t²:0.34]
PhyResSE	–	–	* Se: 42.9 (15.8–75.0) [t²:-] Sp: 100.0 (78.5–100.0) [t²:-]	* Se: 50.0 (9.5–90.5) [t²:-] Sp: 89.5 (68.6–97.1) [t²:-]	* Se: 81.8 (52.3–94.9) [t²:-] Sp: 100.0 (96.3–100.0) [t²:-]	* Se: 81.8 (52.3–94.9) [t²:-] Sp: 99.1 (95.2–99.8) [t²:-]	* Se: 88.9 (56.5–98.0) [t²:-] Sp: 99.0 (94.6–99.8) [t²:-]
SAM-TB	–	* Se: 85.8 (82.3–88.7) [t²:-] Sp: 92.2 (88.7–94.7) [t²:-]	* Se: 86.4 (85.1–87.5) [t²:-] Sp: 97.7 (97.4–97.9) [t²:-]	* Se: 83.6 (76.0–89.1) [t²:-] Sp: 72.6 (67.1–77.6) [t²:-]	Se: 80.0 (76.7–82.9) [t²:0.01] Sp: 99.3 (98.1–99.7) [t²:0.47]	Se: 81.2 (73.5–87.0) [t²:0.10] Sp: 97.4 (96.1–98.3) [t²:0.09]	Se: 75.8 (64.2–84.6) [t²:0.16] Sp: 97.4 (95.6–98.5) [t²:0.16]

^*a*^ Values are presented as follows: Se: Estimate (95% CI) [τ²: Between-study variance], Sp (95% CI) [τ²: Between-study variance]. Se, sensitivity; Sp, specificity; τ² (Tau-squared), the estimated variance of the true effect size across studies (heterogeneity), derived from the bivariate random-effects meta-analysis model. Lower values indicate higher consistency between studies. (*) Asterisk: indicates that a random-effects model could not be fitted due to insufficient data (*N* < 3 studies) or model instability. In these cases, estimates are simple pooled proportions (calculated by summing raw counts across inclusive studies) and do not account for between-study heterogeneity (–, heterogeneity metrics are undefined).

^*b*^ INH, isoniazid; RIF, rifampicin; EMB, ethambutol; PYR, pyrazinamide; STR, streptomycin; FLQ, fluoroquinolones (class); CIP, ciprofloxacin; OFL, ofloxacin; LEV, levofloxacin; MOX/MFX, moxifloxacin; AMK, amikacin; KAN, kanamycin; CAP, Capreomycin.

#### Pooled estimates

For all six evaluated tools (TBProfiler, Mykrobe, MTBseq, GenTB-RF, SAM-TB, and PhyResSE), sensitivity estimates exceed or closely approach the 2021 WHO TPP thresholds for rifampicin and isoniazid (62) (Fig. 2). For rifampicin, TBProfiler achieved a pooled sensitivity of 95.4% (95% CI: 93.5–96.7) and specificity of 97.3% (95.7–98.3), with between-study variance τ² of 0.53 and 0.96, respectively; Mykrobe achieved 93.7% (92.0–95.1) sensitivity and 97.0% (94.8–98.3) specificity. For isoniazid, corresponding estimates were 92.0% (90.4–93.3)/97.3% (96.0–98.2) for TBProfiler and 88.2% (85.5–90.4)/97.5% (95.8–98.5) for Mykrobe.

For ethambutol, the specificity was notably heterogeneous: 87.8% (80.2–92.8; τ² 2.08) for TBProfiler and 92.1% (86.2–95.6; τ² 1.87) for Mykrobe, consistent with previous reports noting its challenging phenotypic classification and common low-grade resistance conferring variants (12). For streptomycin (STR) and pyrazinamide (PYR), performance estimates are more variable between tools. For pyrazinamide, the sensitivity varied from 49.9% (32.5–67.4) for PhyResSE to 80.6% (60.0–92.0) for SAM-TB, generally falling short of the WHO’s targeted 80% sensitivity (62). For streptomycin, a sensitivity-specificity trade-off is apparent, where more sensitive tools (TBProfiler and SAM-TB) are less specific, and vice versa (MTBseq, Mykrobe, and PhyResSE). Sensitivity for key fluoroquinolones levofloxacin and moxifloxacin approaches the WHO-specified 90% threshold (TBProfiler’s sensitivity for levofloxacin is 89.7% [54.7–98.4]), albeit with below-target (≥98%) specificity (91.6% [62.5–98.6]).

Data scarcity for other fluoroquinolones and second-line injectables (Fig. 3) for PhyResSE, MTBseq, SAM-TB, and GenTB precluded obtaining robust pooled estimates.

For other group B and C agents, validation data are scarce, and pooled estimates for sensitivity and specificity are variable and almost universally falling short of the WHO-set sensitivity target of 80% (62).

### Meta-regression

We performed bivariate meta-regression to identify covariates that may affect reported prediction performance. These included the study’s publication year, study size, its multidrug-resistance rate (using rifampicin resistance as a proxy), the proportion of L1% to L9% per study, the source for validation strains (public versus original data sets), geographic origin of strains, and the phenotypic method (MGIT/liquid growth medium versus solid Löwenstein-Jensen medium).

**Fig 5:**
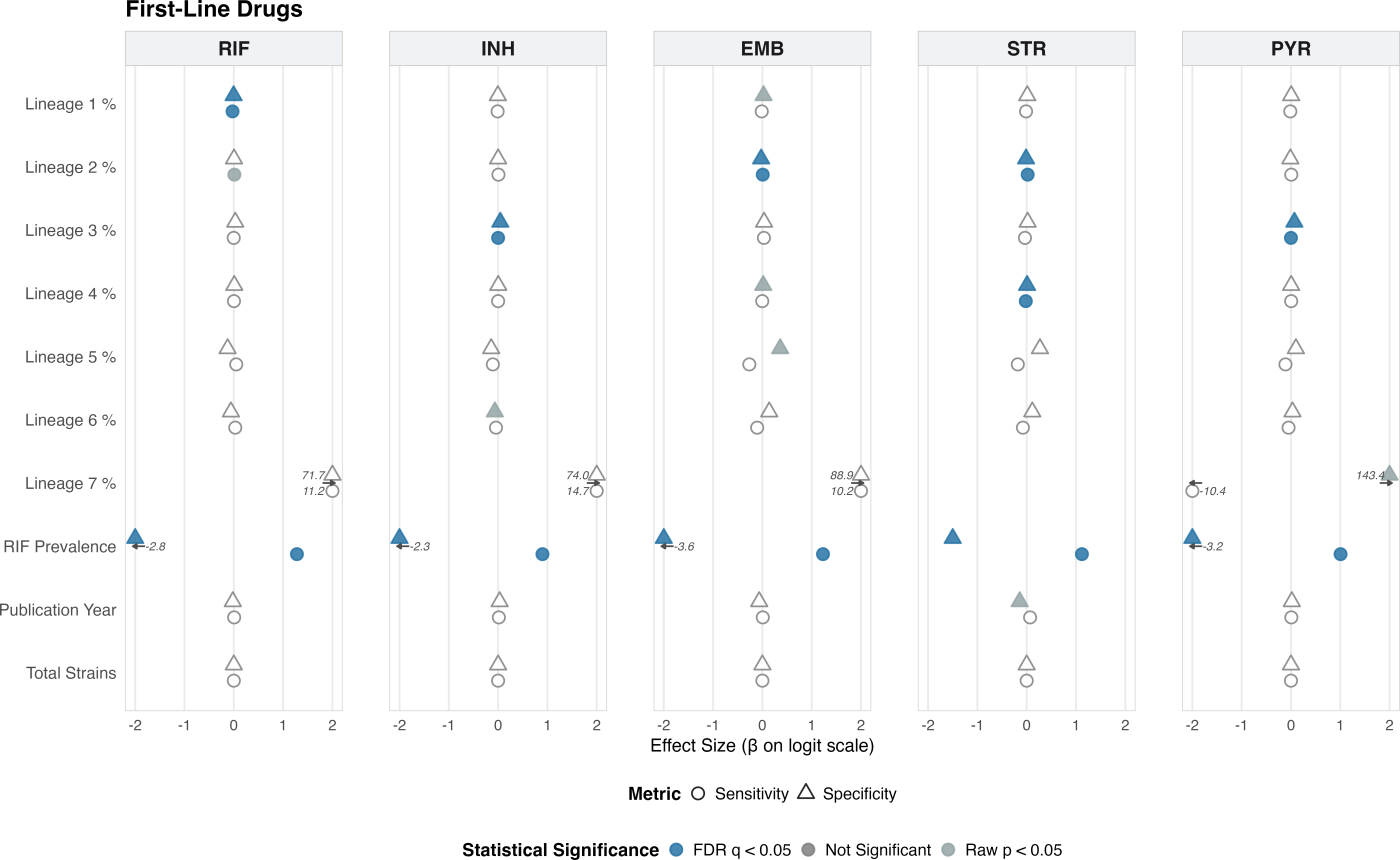
Meta-regression analysis of continuous covariates on diagnostic performance for first-line tuberculostatics and streptomycin. Forest plots showing the effect (ß on logit scale) of lineage proportions, RIF resistance prevalence, publication year, and study size on sensitivity (circles) and specificity (triangles). Filled blue markers indicate significant effects after FDR correction (q < 0.05); filled gray markers indicate nominal significance (*P* < 0.05); hollow markers indicate nonsignificant results. Arrows indicate effect sizes exceeding the plot range. INH, isoniazid; RIF, rifampicin; EMB, ethambutol; PYR, pyrazinamide; STR, streptomycin.

Analyzing covariates pooled by drug, the most significant covariate is a study’s prevalence of rifampicin resistance, which across all first-line drugs predicts a significant decrease in specificity and an increase in sensitivity (*P* < 0.05, FDR q < 0.05, and effect sizes ß −1.5 to −3.6 for specificity and ß 0.9 to 1.3 for sensitivity) (Fig. 5; File S2, and Table S7).

**Fig 6:**
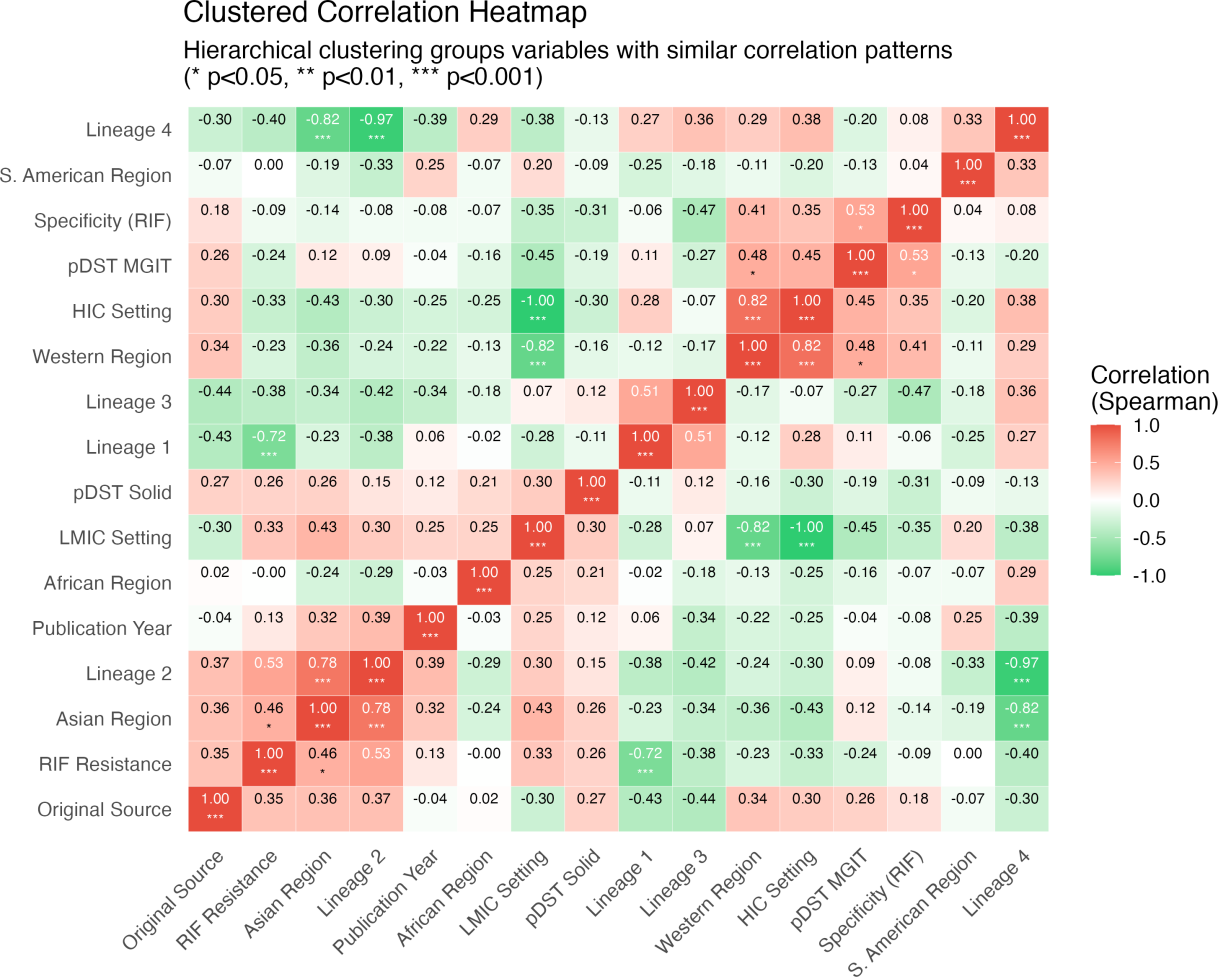
Clustered Spearman correlation heatmap of study-level covariates. Pairwise Spearman’s rank correlation coefficients () were calculated between study characteristics, including resistance prevalence, geographic region, lineage proportions, and publication year. Hierarchical clustering (complete linkage) was employed to group variables with similar correlation profiles. Statistical significance was assessed using a test for association, with *P*-values corrected for multiple comparisons using the Benjamini-Hochberg method (* p_adj_ < 0.05, ** p_adj_ < 0.01, and *** p_adj_ < 0.001). A distinct cluster (bottom-left) reveals strong inter-correlations between RIF Resistance, Asian Region, Lineage 2, and Publication Year, highlighting a confounding structure in the available data. Asian Region includes studies representing China, India, Indonesia, Iran, Japan, Kazakhstan, Taiwan, and Vietnam. Western Region includes studies representing Canada, Finland, France, Germany, the Netherlands, Portugal, and the USA (or labeled broadly as Europe in the source data).

**Fig 7:**
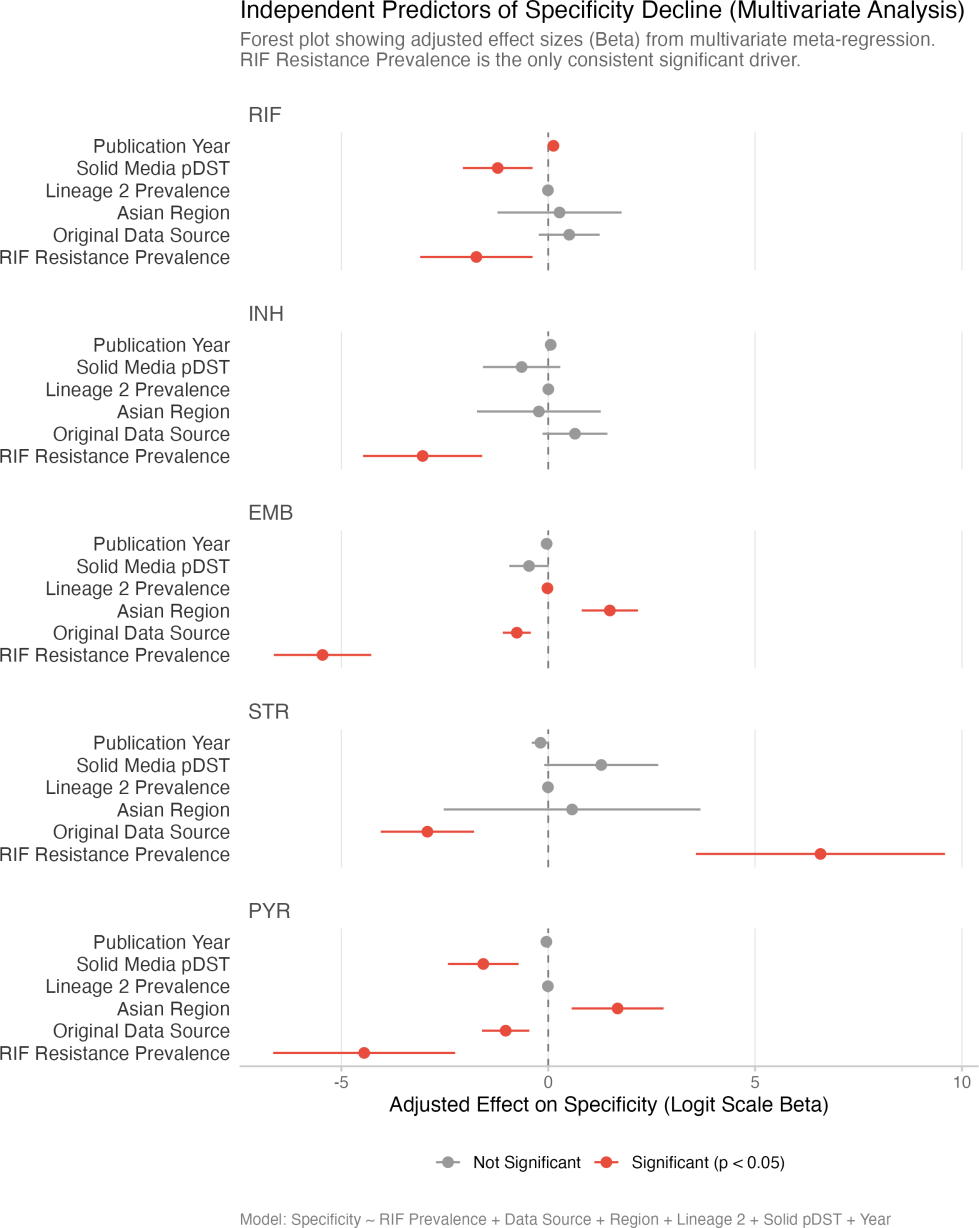
Multivariate predictors of specificity decline. Forest plots showing adjusted effect sizes (Beta coefficients on logit scale) from multivariate meta-regression models. The model adjusts for rifampicin resistance prevalence, data set originality, geographic region (Asia), Lineage 2 proportion, pDST method (solid vs liquid/MGIT), and publication year. Error bars represent 95% confidence intervals. Red markers indicate statistical significance (*P* < 0.05).

Mapping correlations between covariates (Fig. 6), a greater rifampicin resistance co-occurs with L2% predominant data sets, those of Asian origin, and those containing original strains rather than public domain genomes. To clarify which of these four most likely drive the specificity decline, we performed a multivariate meta-regression (Fig. 7; Table S8). Rifampicin resistance prevalence remained the dominant independent predictor of decreased specificity across first-line drugs INH, RIF, PYR, and EMB (*P* < 0.05), with minor additional drivers being originality of the data set (PYR/STR/EMB, *P*-value < 0.001), and the use of solid media pDST for RIF and PYR (RIF *P*-value 0.005 and PYR *P* < 0.001).

In addition to rifampicin resistance, a study’s lineage composition appears as a significant but small-effect size covariate for first-line drugs (correlated with a lower specificity of L1- and L2-dominant data sets and higher specificity in L3- and L4-dominant data sets). Nevertheless, as shown in Fig. 6, L2 prevalence is associated with rifampicin resistance, Asian studies, and original strain sets, and a change-in-estimate analysis showed that most lineage effects are confounded by rifampicin resistance prevalence, and even after adjusting, of negligibly small effect size (Fig. 6 and 7; Table S9).

**Fig 8:**
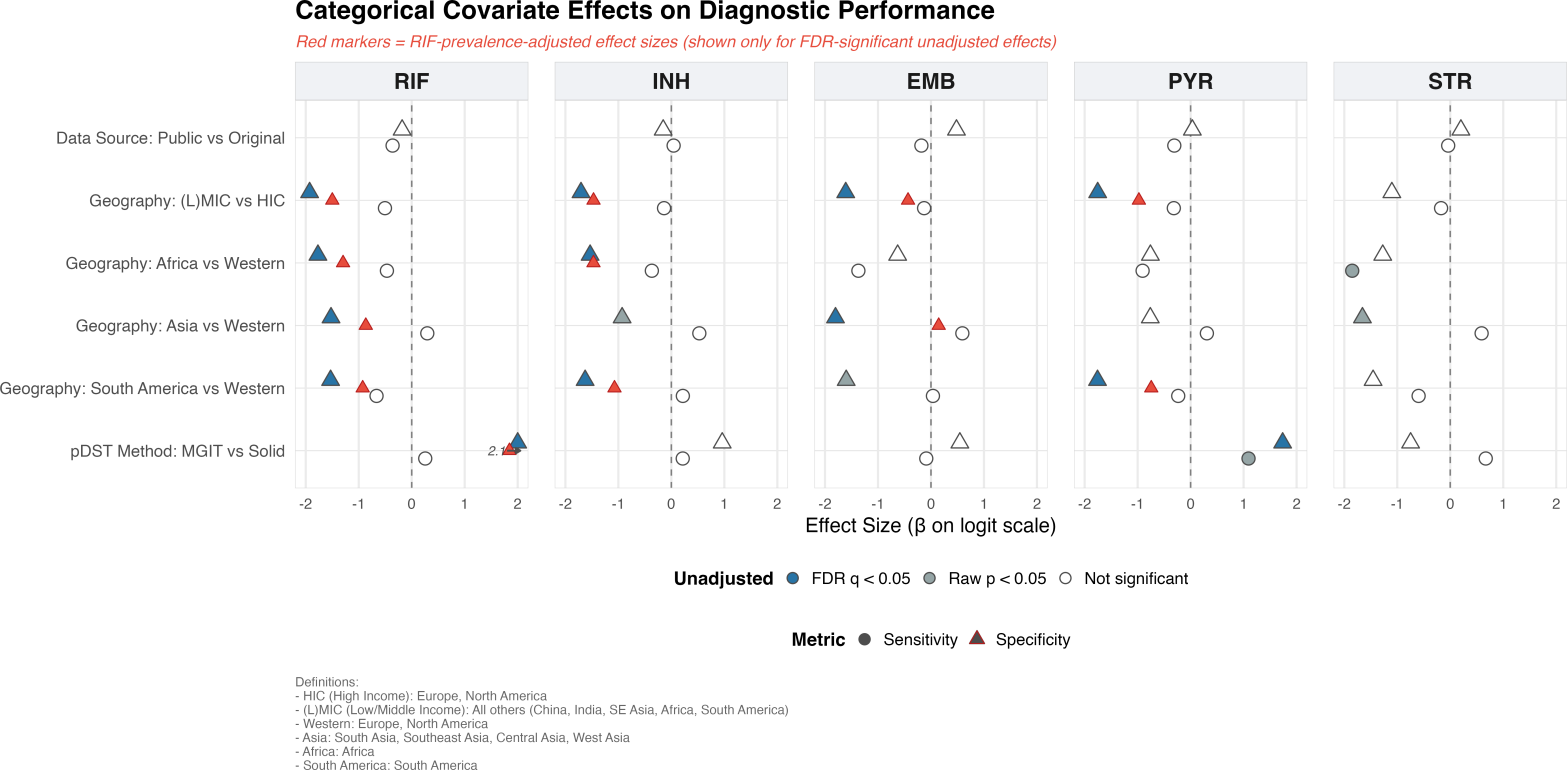
Meta-regression analysis of categorical covariates on diagnostic performance for first-line tuberculostatics and streptomycin. Forest plots comparing effect sizes (ß on logit scale) for the phenotype testing method (reference: solid), data source (reference: original), and geographic region (reference: high-income or Western countries). Circles represent sensitivity effects; triangles represent specificity. Filled blue markers indicate significant differences relative to the reference group (FDR q < 0.05); filled gray markers indicate nominal significance (*P* < 0.05); hollow markers are nonsignificant. Effects > 0 indicate higher sensitivity/specificity compared to the reference. Smaller red markers show the corresponding effect size after adjustment for rifampicin resistance prevalence (shown only for FDR-significant unadjusted effects); attenuation of the red marker toward zero indicates confounding by resistance prevalence. (L)MIC includes both lower-middle income countries and upper-middle income countries. INH, isoniazid; RIF, rifampicin; EMB, ethambutol; PYR, pyrazinamide; STR, streptomycin.

Geographical origin and pDST method (MGIT/liquid versus solid) also appear as significant covariates associated with a specificity decrease for an (L)MIC origin of strains across and specificity increases in studies using MGIT/liquid pDST rather than solid medium pDST (Fig. 8; Fig. S3 and Table S10). However, adjustment for rifampicin resistance prevalence attenuated these categorical specificity effects, with 11 of 29 comparisons losing statistical significance. Notably, the (L)MIC versus HIC specificity effect remained significant and large for RIF (β = −1.50, *P* < 0.001), INH (β = −1.47, *P* < 0.001), and PYR (β = −0.98, *P* = 0.021) after adjustment, while regional comparisons (e.g., Asia, Africa, and South America versus Western) generally lost significance. The pDST effect on RIF specificity (MGIT vs solid, β = +1.84, *P* < 0.001) was also robust to adjustment. These findings indicate that geographic origin and pDST method independently affect reported specificity beyond their correlation with resistance prevalence.

Significant covariates in data stratified by tool-drug pairs and per tool (pooling across drugs) show similar trends as those pooled across all tools (Table S7).

### Risk of bias

**Fig 9:**
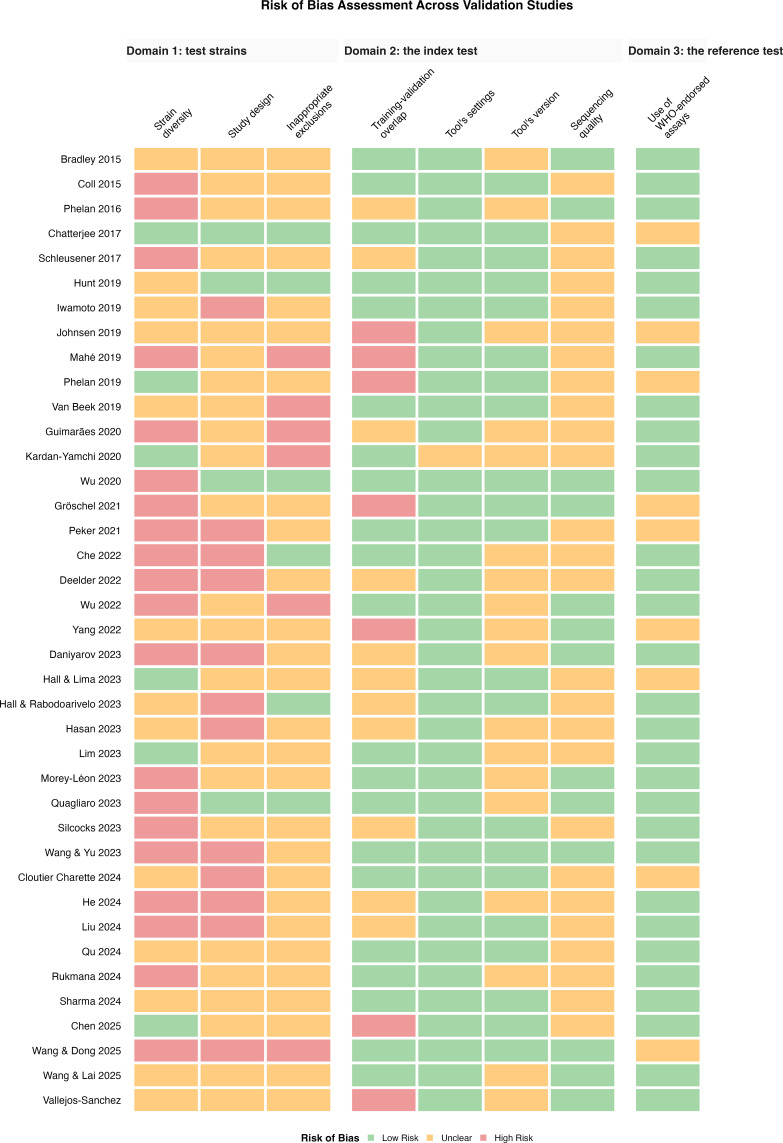
Appraisal of risk of bias in included studies according to the QUADAS-2 tool.

Risk of bias was appraised per study using the QUADAS-2 protocol for evaluation of diagnostic studies (see File S2 and Table 2) (30). Risk of bias was estimated for each of the three categories of variables: those related to the validation data set (selection of genomes), those related to the index test (that is, whole-genome sequencing and the prediction tools used), and those related to the reference test (the phenotypic DST reference). An extended discussion of the appraisal procedure is provided in the File S2, and extracted data are shown in Tables S2 to S4. An overview of risk appraisal is shown in Fig. 9.

In the domain of strain selection, we found that in over half of validation studies (20/39), a single lineage predominates, raising concerns about the generalizability of per-study performance data to other lineages, as raised in other studies (16). We find that sampling procedures for validation sets are often unspecified (23/39 studies), concern case-control sampling (12/39 studies), or are often limited to including only subsets from public data sets (5, 14, 25, 26). This raises inclusion/exclusion bias (e.g., excluding hard-to-phenotype resistance mechanisms or genotypes with known hard-to-interpret variants) and spectrum bias (validating tools on “easy,” high-level resistance phenotypes). In several large-scale studies, we identified likely overlap between the strains used for validating tools and those used for compiling their variant catalogs, raising concerns of “overfitting” and overestimation of performance data.

### Systematic review

We systematically compared features of the meta-analyzed tools in Table S5. To assess the usability of tools in resource-constrained laboratories, we documented for each tool included in the search term features related to portability and accessibility, user-friendliness, clinical usefulness, and parameters of variant calling and variant annotation.

## DISCUSSION

Whole-genome sequencing (WGS) has emerged as the most comprehensive genotypic DST modality available in tuberculosis. Supporting its clinical roll-out, open-access analysis software has become available, offering sample-to-result functionality and promising to “democratize” WGS-based DST for non-experts (5, 6). However, clinical adoption remains hampered by varying reports of software performance, with some studies reporting meeting the WHO’s performance standards (6, 13), whereas others report below-target figures (5, 6, 13, 14, 16, 63). Existing performance summaries are either outdated or limited, at most pooling study data of 8,010 genomes (15, 17), or are focused on internal validity evaluations rather than mapping real-world performance drivers (18). This comprehensive meta-analysis of 144,623 genomes provides an updated summary of tools’ performance and utilizes meta-regression to characterize independent sources of this interstudy heterogeneity.

We found that sensitivity and specificity summary estimates for first-line drugs rifampicin and isoniazid meet or closely approach WHO-specified minimum targets of ≥95%/≥90% and ≥98%, respectively, for all six meta-analyzed tools (TBProfiler, Mykrobe, PhyResSE, GenTB, MTBseq, and SAM-TB), albeit with considerable interstudy heterogeneity (τ^2^ 0.12 to 1.27 for TBProfiler and Mykrobe) (Fig. 2, Table 2). For other drugs, important sensitivity or specificity gaps remain. For ethambutol, specificity is particularly heterogeneous between tools and studies (ranging from 86.5% [SAM-TB] to 95.4% [MTBseq]), whereas for pyrazinamide, sensitivity is less stable than specificity (pooled sensitivity estimates ranging from 49.9% [PhyResSE] to 80.6% [SAM-TB]). This aligns with the known resistance genotype of both drugs: ethambutol is known for low-level resistance-conferring variants (conferring MICs near the critical concentration), commonly translating to genomic false resistant predictions (12, 64). Pyrazinamide is uniquely characterized by a large, heterogeneous array of variants in *pncA*, which are challenging to capture sensitively in resistance catalogs (41, 64). For fluoroquinolones, both sensitivity and specificity estimates closely approach 90% (WHO TPP targets are ≥90%/≥98% for sensitivity and specificity) (62), although a sensitivity-specificity trade-off is apparent (where more sensitive tools lack specificity and vice versa). For “newer” agents (such as bedaquiline and linezolid) and miscellaneous group B and C drugs, data scarcity and lack of resistant phenotypes in validation sets are prominent, precluding any conclusions on genomic prediction performance. These findings highlight several intrinsic limitations of current genomic DST, including dichotomous resistance classification that oversimplifies a quantitative resistance spectrum (EMB and FLQ), inadequate modeling of heterogeneous or complex resistance genotypes (PYR), and the scarcity of genotype-phenotype correlated data for new drugs.

In addition, these estimates must be considered in light of methodological limitations of current validation studies. In our meta-regression, we show that the prevalence of rifampicin resistance is a dominant covariate associated with increased sensitivity and decreased specificity. Several factors may explain increases in reported sensitivity. First, from an epidemiological viewpoint, resistance-conferring variants will tend to amplify clonally, skewing the resistance landscape toward successful variants (which, over time, tend to be well characterized and easier to identify). Second, we identify several sources of selection bias at play in validation studies based on public domain genomes: most do not specify genome sampling procedures or are case-control studies by design, raising concerns about inclusion and spectrum bias (being biased toward including uncomplicated resistance genotypes and phenotypes that are easier to detect). Additionally, several data sets (7, 65, 66) are reused in the training or validation of multiple tools (14, 22–24, 29), creating a risk of “overfitting” of resistance prediction models to particular strain sets. Partial inclusion of public source data sets is common in large studies but creates ambiguity regarding the risk of a possible sampling bias. Studies usually pre-select strains based on the sequencing quality of the genome, albeit using variable inclusion criteria. Studies of genomes with a lower average sequencing depth may result in findings of false genotypic susceptibility since resistance-associated variants in a minority of reads are less likely to be detected. Similarly, genome contamination may cause false positive resistance calls. These variables must be controlled for when extrapolating performance to clinical settings with other sequencing quality/depth targets.

Strikingly, the reported specificity was decreased in high-RIF resistance data sets, which seems at odds with a simple selection bias associated with enrichment of “easy” genomes (i.e., those with both clear-cut resistance and uncomplicated susceptibility). Our multivariate analysis shows that several covariates are at play in causing these specificity declines. Rifampicin resistance rates emerged as the most consistent negative driver, implying that high-resistance-burden settings present a greater challenge to tool specificity, independent of any other variables. Smaller independent drivers include the use of solid pDST media versus liquid/MGIT media (for PYR and RIF) and whether strains were original or re-used public domain strains. Strikingly, lineage composition of validation sets (e.g., predominance of L2 strains in Asian studies) did not significantly affect performance, suggesting that earlier such observations (16) suffered from confounding due to other covariates (such as RIF resistance prevalence). Taken together, these findings suggest that a spectrum bias is the most significant contributor to the reported specificity decline: high-resistance settings likely contain a higher frequency of borderline resistance mutations or complex mixed populations that challenge binary WGS predictions. The importance of sampling bias (i.e., validation studies favoring inclusion of uncomplicated genomes) is relatively smaller but still significant for EMB/PYR/STR, as evidenced by a specificity decline in original studies (which typically contain more consecutive or prospectively sampled strains) versus those assembled from public domain genomes. It should be noted, however, that of 25 studies of original strains, only four explicitly mention prospective, random, or consecutive genome inclusion without inappropriate strain exclusions. Therefore, performance estimates in original-strain studies may still be an overestimation compared to routine clinical practice, which sees the full spectrum of genotypes and phenotypes.

Apart from raw performance estimates, several other considerations affect the clinical utility of these tools. First, estimating clinical performances requires considering the local epidemiology, such as resistance rate, outbreak situations, and the local genotypic resistance landscape. The negative predictive value (NPV) associated with a sensitivity target of 90% is 97.0% at a 20% resistance rate, whereas for a 70% sensitive test, NPV still reaches 91.4%. Therefore, even if pooled estimates may be overestimations, current performance supports using these tools as rule-out tests for rifampicin, isoniazid, ethambutol, and fluoroquinolones. Proprietary bioinformatic resistance prediction pipelines are being used in this capacity by reference laboratories in the UK and Italy (11, 12). Recently, the WHO has specified a more lenient minimum sensitivity target of ≥80% for bedaquiline, linezolid, clofazimine, delamanid, pretomanid, amikacin, and pyrazinamide (62). A target of 98% specificity, WHO’s minimum target, results in a PPV of ~ 90% at 20% resistance rates, whereas a specificity of 90% and 80% results in PPVs of 63.6% and 46.7%, respectively, implying that the tool’s limited specificity remains a critical gap. Nevertheless, in interpreting limited specificity figures, several other factors require consideration. It is well known that pDST is an imperfect standard in TB, which is both technically challenging to execute and imposes an inaccurate binary stratifier to the spectrum of resistance. This is reflected in inconsistently reported pDST results for identical public genomes (67), false susceptible labeling of genomes with canonical resistance-conferring variants (causing half of false resistance predictions for RIF/INH in one study [14]), and false PYR-susceptible labeling of intrinsically PYR-resistant *M. bovis* (observed in 30% of public strains [5]). Answering the challenges of accurate pDST, reference laboratories are increasingly taking up broth microdilution to quantify resistance, using minimal inhibitory concentrations (MICs). Nevertheless, adequate interpretation of genomic DST results still requires familiarity with the phenotypic standard against which results are benchmarked. As an important asset in improving standardization of variant catalogs, several tools (TBProfiler, Mykrobe and successor DrPRG [38]) are now adopting the WHO catalog of resistance-conferring variants, which summarizes the evidence base for all known variants, and introduces expert rules for stratifying variants of unknown significance (64). While this catalog provides a valuable shared framework for variant interpretation, applying it still requires expertise to interpret the mutation grading rules WHO has defined—somewhat undermining the ambition of many open-access tools to fully “democratize” WGS-based resistance prediction.

Based on the findings of this review, we make the following recommendations for future development and evaluation of open-access prediction tools. Since drug resistance burden significantly affects the reported tool’s performance, evaluation studies should be prospective and be conducted in both high- and low-incidence settings, accounting for the full spectrum of samples that are encountered in various clinical settings. If data sets are enriched for resistant phenotypes, these should be balanced to include hitherto underrepresented lineages, such as L5 and L6. Alternative endpoints should be included in future validation studies, including the relation between variants of unknown significance and possible therapy failure and whether detection of a variant is likely to affect decisions related to the treatment regimen (6, 12, 68).

In conclusion, while several WGS-based prediction tools achieve clinically useful accuracy as a rule-out test for first-line resistance, their use as a comprehensive DST modality is currently hampered by data scarcity and limited binary genotypic and phenotypic resistance models. Validation studies show a dominant spectrum bias, which emphasizes the need for prospective studies in high-incidence settings.

## SUPPLEMENTAL MATERIAL

Supplemental materials jcm.00321-26-s0001.docx Files S1 to S3, Tables S1 to S5, and Figures S1 to S3.

Table S6 jcm.00321-26-s0002.xlsx Overview of pooled sensitivity and specificity per drug and per tool.

Table S7 jcm.00321-26-s0003.xlsx Evaluation of study-level predictors of performance using bivariate meta-regression.

Table S8 jcm.00321-26-s0004.xlsx Potential drivers of specificity across first-line drugs.

Table S9 jcm.00321-26-s0005.xlsx Change-in-estimate analysis of categorical variables on model sensitivity and specificity after adjusting for RIF resistance prevalence.

Table S10 jcm.00321-26-s0006.xlsx Change-in-estimate analysis of lineage effects on model sensitivity and specificity after adjusting for RIF resistance prevalence.

ASM does not own the copyrights to Supplemental Material that may be linked to, or accessed through, an article. The authors have granted ASM a non-exclusive, world-wide license to publish the Supplemental Material files. Please contact the corresponding author directly for reuse.
