# Supplementary material for: Open-access genomic drug resistance prediction tools for Mycobacterium tuberculosis: a systematic review and meta-analysis
Source: J Clin Microbiol. 2026 Jul 27;64(8):e00321-26. doi: 10.1128/jcm.00321-26 (PMC13463831; doi:10.1128/jcm.00321-26)
Supplement: Supplementary file 1 [file jcm.00321-26-s0001.docx]

Online Supplement to **‘Open-access genomic drug resistance prediction tools for *Mycobacterium tuberculosis*: a systematic review and meta-analysis’**, Dewaele K, Jouego C, Asim A, Laenen L, Meehan C, André E.

Table of Contents

[Supplementary File S1: Search strategy	2](#_Toc233025966)

[Supplementary File S2. Adapted QUADAS-2 guidelines	4](#_Toc233025967)

[Supplementary File S3. Narrative review of genomic prediction tools	14](#_Toc233025968)

[Supplementary Table S1. Methodology of data extraction per study.	22](#_Toc233025969)

[Supplementary Table S2. Extracted quality control data for Domain 1	27](#_Toc233025970)

[Supplementary Table S3. Extracted quality control data for Domain 2	51](#_Toc233025971)

[Supplementary Table S4. Extracted quality control data for Domain 3	71](#_Toc233025972)

[Supplementary Table S5. Systematic review of genomic prediction tools	78](#_Toc233025973)

[Supplementary Figure S1: Diagnostic performance of whole-genome sequencing (WGS) tools for miscellaneous group A, B and C drugs.	80](#_Toc233025974)

[Supplementary Figure S2. Meta-regression analysis of continuous covariates on diagnostic performance for fluoroquinolones and second-line injectables.	81](#_Toc233025975)

[Supplementary Figure S3. Meta-regression analysis of categorical covariates on diagnostic performance for fluoroquinolones and second-line injectables	83](#_Toc233025976)

[References	84](#_Toc233025977)

# Supplementary File S1: Search strategy

To develop a comprehensive search term for our meta-analysis, we first aimed to compile a representative list of available genomic prediction tools for *Mycobacterium tuberculosis* drug susceptibility.

First, we identified a set of established tools known for their for their **foundational contributions** or frequent use in the field, and that are frequently included in major benchmarks. (1–8) These included TBProfiler, Mykrobe, CASTB, KvarQ, TGS-TB, PhyResSE, MTBseq, and ReSeqTB/ReSeqWHO. We then performed a citation-chaining search using the key publications for the most-cited tools. (1,3,9–12). Articles citing these key publications were screened to identify more recently developed tools with the same drug-susceptibility prediction functionality.

This yielded 14 additional public WGS-based drug-resistance prediction tools (Table S1.1), excluding pipelines with partial drug-prediction functionality (for example, variant callers like XBS (13) and binoSNP (14) or annotation-only algorithms, like MD-CNN (15)), and tools designed for genotype-phenotype association discovery rather than clinical decision support (for example, funTB (16) and Kover (17)). Further, to focus our meta-analysis on tools with proven community adoption, we excluded 10 tools that appeared in only a single publication.

Together, this yielded a search term comprising 13 commonly used tools, which was used to query Pubmed, Embase and Web of Science databases on Oct 29th 2025:

(“mykrobe” OR “tbprofiler” OR "tb profiler" OR “tb-profiler” OR “castb” OR “kvarq” OR “phyresse” OR “tgs-tb” OR “tgstb” OR “mtbseq” OR “mtb-seq” OR “reseqwho” OR “reseqtb” OR “gentb” OR (“magma” AND “tuberculosis”) OR (“pointfinder” AND “tuberculosis”) OR “sam-tb” OR “samtb”)

**Table S1.1. Identified genomic drug-susceptibility prediction tools in order of decreasing citations.**

| **Tool Name** | **Publication year** | **Records in Pubmed**  **(Oct 2025)** |
| --- | --- | --- |
| **TBProfiler** (3,10) | 2015 | 77 |
| **Mykrobe** (1,9) | 2015 | 46 |
| **PhyResSE** (12) | 2015 | 21 |
| **MTBseq** (11) | 2018 | 19 |
| **KvarQ** (18) | 2014 | 9 |
| **ReSeqTB** (19) | 2018 | 9 |
| **SAM-TB** (20) | 2022 | 9 |
| **CASTB** (21) | 2015 | 8 |
| **MAGMA** (22) | 2023 | 8 |
| **TGS-TB** (23) | 2015 | 7 |
| **GenTB** (7) | 2021 | 5 |
| **PointFinder (for Mtb)** (24) | 2019 | 2 |
| **BAGEP** (25) | 2020 | 1 |
| **Resistance Sniffer** (26) | 2020 | 1 |
| **Sciensano-Galaxy pipeline** (27) | 2021 | 1 |
| **COMBAT-TB Workbench** (28) | 2022 | 1 |
| **MycoVarP** (29) | 2022 | 1 |
| **DrPRG** (30) | 2023 | 1 |
| **AAICare®-TB** (31) | 2024 | 1 |
| **GenoMycAnalyzer** (32) | 2024 | 1 |
| **TB-DROP** (33) | 2024 | 1 |
| **TB-AMRpred** (34) | 2025 | 1 |

# Supplementary File S2. Adapted QUADAS-2 guidelines

The process of appraisal of study quality is described here. QUADAS-2 is a protocol for the evaluation of diagnostic study quality recommended by the Cochrane Collaboration (35). Here, we adapt the QUADAS-2 tool to address sources of bias encountered in diagnostic studies of genomic prediction tools (36).

QUADAS-2 stipulates four key domains, with each containing several signalling questions for evaluation of the risk of bias and of applicability of diagnostic studies.

1. **Domain 1: patient selection**

The QUADAS-2 guidelines provide examples of signalling questions for estimating the risk of bias. These questions are the following:

- Was a consecutive or random sample of patients enrolled?
- Was a case-control design avoided?
- Did the study avoid inappropriate exclusions?

As explained further, we adapted these questions to the particularities of diagnostic studies of genomes.

1.1 *Signalling question 1: Was a consecutive or random sample of patients enrolled?*

***Adapted question****: What is the risk of sampling bias due to the use of phylogenetically monotonous strains?*

Generally, random or consecutive enrolment is desirable to avoid a sampling bias. In the context of genomic prediction software, performance is determined by how well the tool detects resistance-conferring variants in sequencing data. Thus, performance representativeness will be affected by how well resistance-conferring variants in the test population represent those in the target population. Resistance prediction tools should generalise across the diversity of resistance mechanisms found in global strains. However, random inclusion of clinical strains may not necessarily improve this representativeness. Particularly in tuberculosis-endemic areas, clinical strains tend to be clonal, resulting in monotonousness of resistance-associated variants. Since resistance mechanisms vary by phylogenetic background (4,10), we consider phylogenetic diversity a more appropriate metric of representativeness than random strain inclusion. Consequently, for this signalling question, we assessed representativeness primarily using the test population’s phylogenetic composition.

To assess the risk of a study having limited global representativeness, we considered a study at high risk of bias if over 50% of included strains belonged to a single lineage. Risk was scored ‘unclear’ if no information on the distribution of lineages was given.

Additionally, we recorded the number of rifampicin-resistant or multidrug-resistant strains when reported. A larger proportion of resistant strains may indicate a greater diversity of resistance mechanisms, and thus greater global representativeness. Since the diversity of resistance mechanisms is also determined by other factors (clonality, the total size of the dataset), resistance rates were recorded but not used in scoring the risk of bias.

1.2 *Signalling question 2: Was a case-control design avoided?*

***Adapted question****: What was the risk of sampling bias inherent to the study design (a case-control versus a prospective study design)*

A case-control design carries an increased risk of various biases. In studies of gradual phenomena such as drug resistance, this may particularly result in a risk of spectrum bias: inclusion of samples classified in a binary manner risks limiting applicability to real-world practice, where categories may be non-binary. Drug resistance is a non-binary phenomenon that spans degrees, as reflected in clinical categories such as ‘low-level’ and ‘high-level’ resistance (37).

Spectrum bias stemming from quantifying resistance as a binary phenomenon is an intrinsic limitation of phenotypic DST in current genomic prediction studies (see Domain 3, Supplementary Table S4). Phenotypic DST mostly relies on binary criteria, namely critical concentrations. This bias may be aggravated in studies that only include isolates for which phenotypic testing results were unambiguous (that is, high-level resistant or clearly susceptible strains), such as is done in case-control study designs, rather than including strains from the complete resistance spectrum.

The risk of bias due to study design was assessed as follows. Studies that mentioned a case-control design were scored at high risk for bias. Studies that mention using a prospective dataset without sample exclusions (indicating that all strains collected in a given period were included) were rated at low risk for bias. For all other studies risk was considered unclear.

1.3 *Signalling question 3: Did the study avoid inappropriate exclusions?*

This signalling question was retained unchanged. In addition to a sampling bias resulting from study design, bias may stem from inappropriate strain exclusions.

Validation studies that assemble datasets consisting of public strains may be vulnerable to exclusion bias if datasets are not included in their entirety. Indeed, a comparison of isolates included in evaluation studies and isolates available in source datasets shows partial inclusion of source datasets in several large studies (e.g. (3,9,10)). Reasons may vary, including, for example, varying requirements between source and evaluation studies regarding sequencing depth. We assume that inadequate sequencing quality is a random variable that does not confer a risk of bias. However, other reasons for incomplete inclusion may confer bias, such as the exclusion of strains with complex or ambiguous resistance patterns, strains with discordant genotype-phenotype relationships, or strains that perform poorly with the prediction algorithm. Since partial exclusion or reasons for exclusion are typically not discussed, the risk of bias was scored as unclear for all studies that included source datasets in part. Studies in which mention was made of sample exclusions were rated at high risk of exclusion bias, except for the exclusion of samples due to random variables (e.g. those with inadequate raw sequencing data quality). If no mention was made of sample exclusions and the dataset consisted of original strains that were included consecutively or randomly, it was assumed that no inappropriate exclusions had taken place, and risk for bias was rated low.

Finally, QUADAS-2 includes an assessment of applicability, which examines whether a study aligns with real-world clinical scenarios, as expected during clinical use of drug-resistance tools. However, beyond the concerns related to sampling bias already addressed through the three signalling questions, we consider that the procedure of studying drug resistance using WGS in validation studies accurately reflects the conditions of the tool’s clinical use. We therefore have no other applicability concerns.

Each study is appraised for these variables in Supplementary Table S2.

2. **Domain 2: the index test**

For domain 2, the index test, several signalling questions are proposed in the QUADAS-2 tool, which we adapt as specified:

2.1 *Signalling question 1: Were the index test results interpreted without knowledge of the results of the reference standard?*

***Adapted question****: Were the training strains different from the validation strains?*

None of the included studies reported researcher blinding for results of either the index test or the phenotypic reference test. This is considered of less importance for WGS-based and phenotypic DST studies, since interpretation of both is assumed to be observer-independent (involving the use of prespecified thresholds or breakpoints).

This question was adapted to reflect the risk of overestimation of diagnostic performance in the case that a set of genomes was used for validation of the performance of a genomic tool that was also used to train this tool.

If strains in a validation set had also been used to train the software tool to be validated, the risk of bias was scored high. Risk was scored low in case validation and training strains were mentioned to be different, or when these could be manually determined to be different. In other cases, the risk was rated unclear.

2.2 *Signalling question 2: If a threshold was used, was it pre-specified?*

***Adapted question****: What is the risk of measurement bias due to 1) variable tool settings, 2) variable tool versions, and 3) variables in whole-genome sequencing?*

The use of variable tool analysis parameters may result in a measurement bias between studies. By the design of this meta-analysis, only studies that used the prediction tools in their default configuration were included; that is, studies that used modified tools or parts of the tool (e.g. its variant calling pipeline or resistance mutation catalogue) were excluded. However, studies that used prespecified operation modes of a tool were not excluded; for example, MTBseq may be used in its low-frequency variant detection mode, as applied by Kardan-Yamchi et al., 2020, and for Mykrobe, various parameters may be changed (38,39). Since this nevertheless might result in bias, we documented per study what settings were used and scored bias as unclear if non-default settings were used. Notably, most studies do not explicitly mention the use of the default settings of a tool. However, since user-adaptability is generally limited and adaptation is not typically required for use, we reasonably assumed that when no changes to settings were mentioned, default settings were used.

Due to rapid developments in the field, comparisons of tools’ performance may be biased by the use of outdated versions. We documented the mentioned versions per study and scored the risk of bias as unclear if any of the tool’s versions were not mentioned. For meta-analysis, performance data were stratified according to the tool’s versions.

Sequencing variables may affect variant calling and, therefore, the detection of resistance-associated variants.

Several studies demonstrate good reproducibility between different Illumina platforms and chemistries, and between IonTorrent, Oxford Nanopore and Illumina-generated sequences (1,3,40). Therefore, sequencing technology was in itself not considered a risk factor for bias. Strains included in considered studies were almost all sequenced using Illumina technology.

The use of genomes of variable average sequencing depth and coverage may result in decreased sensitivity for detection of minority variants and of variants in low-coverage regions of the genome, respectively (as shown by, for example, (7)). Beyond exclusion of genomes deemed of insufficient quality from the study set by the researcher, prediction tools typically integrate quality-control filters and threshold for variant calling, which affects variant calling. Since these are intrinsic characteristics of the tool, our aim was to assess a risk of bias due to exclusion of genomes by the researcher.

Requirements for read depth and genome coverage of strains in the dataset are variably reported, either as ranges of median or mean read depths per included genome, or as a required read depth for variant calling. Genome coverage is variably reported as coverage for 1x or another fold read depth. We assumed that the variability in thresholds used for these parameters reflects that seen in clinical practice. Extrapolation of performance from a given dataset to clinical practice should take into account the limitations imposed by the reported depth and coverage. Therefore, we quantified the risk of bias based on the clear reporting of these parameters (depth and coverage). When this was not or ambiguously reported, risk was rated unclear. In other cases, risk of bias was rated low.

We did not identify concerns related to applicability of the modalities of the index test used in validation studies and those used in clinical practice.

Each study is appraised for these variables in Supplementary Table S3.

3. **Domain 3: the reference standard**

The QUADAS-2 tool comprises an estimation of the risk of bias stemming from the reference standard, its conduct, or its interpretation.

3.1 *Signalling question 1: Is the reference standard likely to correctly classify the target condition?*

*Adapted question: were WHO-endorsed assays for phenotypic DST used?*

As discussed in the main text, phenotypic DST is a suboptimal reference test for several tuberculostatics, including rifampicin, pyrazinamide and ethambutol, also when WHO-endorsed assays are used (41–43).

This concerns an intrinsic limitation of drug-susceptibility testing in Mtb. To assess source of bias beyond this intrinsic limitation, we documented whether studies reported on the phenotypic reference tests used, and whether these concerned WHO-endorsed assays and standard procedures. If this was the case, risk of bias was rated low. When this information was not available, risk of bias was rated unclear. When non-standardised assays were used, bias was rated high. As an exception to this rule, novel reference assays, such as broth microdilution (the well-validated UKMYC5/6 broth microdilution plate and Thermo Fisher TREK Sensititre MYCOTB), have recently been considered for WHO-endorsement, but have been used as robust reference standards in research settings for a longer time.(44,45) Use of these was not considered to convey a risk of classification error.

3.2 *Signalling question 2: Were the reference standard results interpreted without knowledge of the results of the index test?*

As mentioned, none of the included studies reported researcher blinding for results of either the or the phenotypic reference test. This is considered of less importance for WGS-based and phenotypic DST studies, since interpretation of both are assumed to be observer-independent (involving the use of prespecified thresholds or breakpoints). This signalling question was therefore not retained.

We did not identify concerns related to applicability of the reference test used in validation studies and those used in clinical practice.

Each study is appraised for these variables in Supplementary Table S4.

4. **Domain 4: flow and timing**

QUADAS-2 recommends the assessment of the risk of bias that could have resulted from timing or process variables. The recommended signalling questions are the following:

4.1 *Signalling question 1: Was there an appropriate interval between index test and reference standard?*

Ideally, the index test and reference test are performed at the same time, from the same culture, which is typically the case for prospective studies. However, retrospective studies rely on stocked MTB isolates, for which phenotypic DST was performed at an earlier time than whole genome sequencing. Since in stocked isolates, in the absence of selective pressure, *de novo* acquisition of resistance-conferring is unlikely, the interval between pDST and genome sequencing was considered an unlikely source of bias. Therefore, risk was considered low for all studies.

4.2 *Signalling question 2: Did all patients receive the same reference standard?*

It is possible that genome datasets are enriched for strains with hard-to-interpret phenotypes, which could have prompted the use of sequencing to resolve observed phenotypic discrepancies. This would confer the risk of evaluating tools in a dataset containing an increased proportion of strains with more complicated resistance mechanisms, and therefore possibly underestimate performance. All studies with a non-prospective design would be vulnerable to such a bias. Since study design as a source of bias has been appraised in Domain 1, this is not appraised separately.

4.3 *Signalling question 3: Were all patients included in the analysis?*

In the context of WGS studies of MTB strains, this question is considered identical to Signalling question 3 of Domain 1 and is therefore not appraised separately.

# Supplementary File S3. Narrative review of genomic prediction tools

1. ***Characteristics of unmaintained tools***

Considered here are tools of which the last public software update is more than five years before the search date of this systematic review, the 29th of October 2025.

Introduced in 2014, KvarQ was among the first public DST prediction tools on the scene (18). Unusual among prediction tools, it omits mapping of reads against a reference genome. Predictions are based on direct comparison of raw reads to a set of variant sequences known to confer resistance. Although computationally efficient, this algorithm does not detect resistance-conferring structural variants (insertions or deletions) and, generally, does not allow detection of variants other than those defined in the reference library. Therefore, novel variants in resistance-associated regions of the genome will not be detected.

A complementary web server-based tool, TGS-TB (‘Total Genotyping Solution for TB’), relies on KvarQ’s lineage classification and DST algorithm, and adds software implementations of conventional mycobacterial typing schemes (IS6110 Restriction Fragment Length Polymorphism typing (RFLP), spacer oligonucleotide typing (spoligotyping) and VNTR typing) (23). Using the H37Rv genome as a reference, it also detects core-genome SNPs, enabling detailed strain typing studies. Development of KvarQ’s DST algorithm ceased after 2014. The TGS-TB server is presently not any longer accessible (46).

CASTB or ‘Comprehensive Analysis Server for Mycobacterium Tuberculosis complex’ is a web server that implements an algorithm for lineage determination, in addition to several typing schemes, and a drug-susceptibility prediction algorithm (21). The tool’s authors provide no details on its susceptibility prediction algorithm. Also, the tool does not report the genetic basis for its susceptibility predictions (5). Use of this tool is restricted to academic users after registration.

2. ***Characteristics of recently maintained tools***

PhyResSE (‘Phylo-Resistance Search Engine’) was introduced as a web server that comprehensively reports analysis and quality control parameters (12). Use of a whole-genome mapping algorithm enables detection of different types of genetic abnormalities that are associated with resistance. PhyResSE reports genome-wide variants and predicts drug susceptibility and MTB lineage. Variant Call Format (VCF) and FASTA files may be exported for further phylogenetic and susceptibility studies with external software.

The ReSeqTB platform was developed in 2018 by an international consortium of experts as an effort in standardizing WGS-based drug-prediction. A public web server was developed consisting of a variant calling pipeline and a knowledgebase, containing a curated dataset of correlated genetic and phenotypic susceptibility data (19). Innovative features included an improved algorithm for detection of long deletions and confidence-graded reporting of variants. The platform was transferred under WHO authority in 2019, but, due to funding shortages, was shut down prematurely early in 2021 (47). Only a single publication has reported on ReSeqTB’s performance for drug-susceptibility predictions (19).

MTBSeq was developed in 2018 as a locally installed bio-informatics pipeline, consisting of independent modules for complete genome mapping, variant calling, variant annotation, phylogenetic classification and quality control (11). Its modular design permits control over each step of the analysis. In addition to drug susceptibility prediction, it determines strain lineage, species determination within the *Mycobacterium tuberculosis* complex, and may output FASTA files of aligned alleles and SNP distance matrices of sets of strains for detailed phylogenetic analysis. Operation requires command-line experience. In a benchmark, MTBseq was significantly slower and computationally more demanding than competitor tools (1). Its working memory requirements of 20-25 GB exceed what is typically available on ordinary personal computers.

User-friendliness and portability has been a strength of two early tools that are under active development, Mykrobe (earlier named Mykrobe Predictor, introduced in 2015) and TBProfiler (introduced in 2015). Both are computationally light-weight and offer a convenient user interface; Mykrobe as an offline graphical user interface application, TBProfiler as a web server with a user-friendly web interface (both in addition to availability of a command-line interface) (9,10). Both can analyse data delivered by the portable Oxford Nanopore Technology (Oxford, U.K.) sequencers (1,3).

For variant calling, Mykrobe employs a custom algorithm that does not rely on mapping of reads. Instead, it constructs a de Bruijn graph (a compact mathematical representation of a nucleotide sequence) of the sample and compares it to a reference de Bruijn graph that contains a set of known resistance- and susceptibility-associated variants (9). This lightweight algorithm may be run on smartphones or low-cost single-board computers (1). However, similarly to KvarQ, Mykrobe does not detect novel variants of uncertain significance. In addition to predicting drug resistance and lineage, Mykrobe implements a well-validated algorithm for both *M. tuberculosis* complex and nontuberculous mycobacterial species identification (1). Mykrobe does not support studies of transmission based on SNP distance.

TBProfiler maps reads to the H37Rv reference genome, but only for regions known to be involved in drug-resistance (so-called ‘stamp mapping’). This allows the application to be computationally lightweight and yet able to detect novel, potentially resistance-conferring variants in mapped regions of the genome. In addition, TBProfiler determines the strain’s lineage, spoligotype, and Region of Difference (RD) group, and can optionally generate Newick files for phylogenetic tree reconstruction.

GenTB is a webserver and command-line tool for drug-susceptibility prediction, introduced in 2021. Among the tools reviewed here, it is the only tool that employs machine learning algorithms to model resistance, implementing a random forest-based algorithm and a wide-and-deep neural network for its predictions (7). GenTB’s random forest-based model reportedly offers better accuracy for predicting second-line drug susceptibility than the neural network, and the neural network provides higher prediction accuracy for first-line drugs (7). In addition to predicting susceptibility profile and lineage, the GenTB website comprises a database where users can share correlated genotype-phenotype data.

ResFinder/PointFinder is a web platform and command-line tool supporting genomic DST prediction in multiple bacterial species. ResFinder was originally developed to identify horizontally acquired resistance genes, whereas PointFinder targets species-specific chromosomal mutations.(48) Matching variants in sequencing data with a reference catalogue is done using BLASTn. A recent version introduces indel-based predictions.(24) Only a single independent publication has reported on the tool’s performance.(49)

SAM-TB is an online tool co-developed by Fudan University, the Chinese Antituberculosis Association and the governmental Center for Disease Control in Shenzhen (20). It provides genomic drug-susceptibility prediction, species identification and strain-relatedness analysis.

3. **Features of resistance catalogue and annotation**

In a comparative study of CASTB, KvarQ, Mykrobe Predictor TB and PhyResSE, performance differences were largely explained by the comprehensiveness of tools’ mutation catalogues (5). The comprehensiveness of resistance catalogues directly affects prediction performance. For pyrazinamide, for example, recent studies have uncovered a large number of previously unknown resistance-conferring variants, greatly increasing the sensitivity of prediction algorithms in recent years (1,7,50).

Accuracy of predictions also depends on annotation algorithms, particularly for variants with unknown or a low-grade effects on phenotype. The inclusion in catalogues of variants with a low-grade or ambiguous relationship to phenotypic resistance, can be done in the interest of optimizing sensitivity for detecting resistance. This is illustrated by a performance comparison between the 2019 TBProfiler catalogue and the 2018 CRyPTIC Consortium pipeline, in which TBProfiler exhibited improved sensitivity for all first-line tuberculostatics, at the cost of a minor specificity loss for ethambutol and pyrazinamide (in the order of 1%) (3,51).

The effect on phenotype of ambiguous variants can also be modelled using more sophisticated annotation algorithms. Tools have traditionally used ‘direct-association’ algorithms, where phenotypic resistance is binarily inferred from the presence of variants known to be associated with phenotypic resistance. However, annotating complex resistance mechanisms, including those involving epistasis (synergistic or antagonistic interactions between variants), may require more sophisticated models (7).

As a first way of dealing with the non-binary phenotype that results from certain resistance mechanisms, a system of ‘confidence grading’ of variants was proposed.^36^ This consists of assigning a diagnostic likelihood ratio to each variant. Roughly, this value is based on the ratio of the prevalence of the mutation in a resistant population, and its prevalence in a susceptible population. By convention, likelihood ratios > 10 represent a strong association between the presence of a variant and a resistant phenotype (19). A confidence-graded approach was first applied in the now-retired ReSeqTB/ReSeqWHO tool.

More recently, multivariate prediction approaches offer advantages over direct-association based prediction, since these can model between-variant interactions. They are capable of modelling both single SNP-based, binary phenotypes, as complex, polygenic resistance phenotypes. Others have explored a variety of other types of machine-learning models for predicting drug susceptibility in MTB (52–54). Remarkably, advanced machine-learning-based models have delivered similar, or at the most, marginally improved performance compared to best-performing direct-association algorithms, as also is the case for GenTB (7,52). This suggests that the relative importance of polygenic resistance mechanisms is minor in the used validation datasets. Nevertheless, as we identify in the quality appraisal of included studies, a risk of spectrum bias might result in an overrepresentation of simple resistance mechanisms, possibly contributing to the observed limited added value of multivariate models.

4. **Features of variant calling pipelines**

In addition to variant annotation, variables in variant calling pipelines may also contribute to widely different predictions between tools. For example, Mahé et al. find that, in a single dataset, the same variant may be detected in up to ten times more strains with one tool versus another.^1^ Hetero-resistance may complicate the process of variant calling. Due to the error rates intrinsic to sequencing technologies, the use of thresholds is required for differentiating likely sequencing errors from minor variant populations present in the sample. However, frequency thresholds have varied between tools and, for the same tools, between studies (5% by Kardan-Yamchi et al. using MTBseq low-frequency mode (38), 40% by Gröschel using GenTB (7), and 75% for MTBseq with its default settings) (11). Additionally, there is evidence that allele frequency thresholds may require optimisation per drug. For example, Hunt et al. show that optimal minimum frequency thresholds for second-line drugs may be lowered to 1-10%, whereas such low thresholds increase false-positive calls for first-line drugs (1,4).

Additional variables such as read trimming, duplicate read removal and realignment around insertions and deletions may also affect allele frequencies; these operations are inconsistently used among different prediction tools (see Supplementary Table S5) (5,55). Finally, the number of reads covering an allele and base call phred quality scores are significant parameters for which applied thresholds vary widely. All these variables represent the many levels at which validation should be performed, in order to obtain standardised, reproducible and accurate genomic drug-susceptibility predictions.

5. **Features regarding analytic transparency, record-keeping and data protection**

A requisite for clinical implementation concerns compliance with regulations regarding analytic transparency, record-keeping and patient data privacy (5,6,56). The advent of CE-IVD regulations would require software tools to comply with rigorous requirements in this area. However, in their current format, tools are not adapted to clinical implementation. All tools are released with a research-use-only disclaimer, and features for efficient integration in diagnostic workflows are lacking (for example, tracking of sample metadata, logging of user actions, availability of an application programming interface (API) for communication with the laboratory information system). Standardised and reproducible use of analysis pipelines would require transparency regarding software versions and parameters, including those of the tool’s individual components (those for mapping and variant calling, for example, which often have been developed by third parties). Such comprehensive version reporting is not provided by any of these tools. For graphical user interface applications or webservers, even more than for command-line tools, information on versions and settings of individual software components is non-transparent. Standardization is further impeded by variability in versions of the tool’s ‘dependencies’ (that is, external software on which the tool depends to run correctly). For their installation, almost all tools make use of a software ‘environment manager’ such as Conda (Anaconda Inc, TX, USA) that analyses and regulates mutual compatibility of software components to be installed. However, this process often allows for the installation of any of a series of different software versions of each component, depending on pre-existing installations and their mutual compatibility. Complete version reporting of the tool, its dependencies and its mutation catalogue, is therefore of the utmost importance for providing reproducible analyses.

The use of remote webservers (the case for GenTB, PhyResSE, and others) may pose problems with regards to data protection and patient privacy. Transfer of patient-related data over the internet would require the use of secure file transfer protocols; use of these protocols is not specified for most web server tools surveyed here (PhyResSE, for example, is an exception) (5). The potential presence of human DNA in next-generation sequencing data would require stringent privacy protection measures, including removal of any personally identifiable information (including human genetic reads) before manipulation of data in non-protected environments (such as servers hosted by non-clinical organizations). With regards to data storage and ownership, privacy disclaimers of tools are silent or opaque regarding who has access to submitted genomes, the duration of data storage, and eventual reuse of data by maintainers of the tool.

Therefore, clinical use of these tools requires significant improvements with regards to connectivity to diagnostic workflows, transparent version control, and compliance to data protection and privacy regulations.

# Supplementary Table S1. Methodology of data extraction per study.

|  | **Methodology and sources for data extraction** |
| --- | --- |
| **Bradley 2015** | FN, FP, total resistant and sensitive strains, VME and ME rate were extracted from Supplementary Table S9 and S10 for Mykrobe Predictor and KvarQ respectively. TN and FN were calculated. |
| **Chatterjee 2017** | TP, TN, FP, FN for Mykrobe Predictor were counted in Table 2. Performance data for PhyResSE was not included in the meta-analysis, since PhyResSE was only used for 9 strains to resolve discrepancies between Mykrobe Predictor and pDST. |
| **Che 2022** | TP, FN, FP, TN were extracted from Table 2. |
| **Chen 2025** | TP, FN, FP, TN were calculated from Additional File 1. |
| **Cloutier Charette 2024** | TP, FN, FP, TN were calculated from Table 1. |
| **Coll 2015** | Table 2 reports sample size, number of resistant strains, sensitivity and specificity per drug: TP, FP, TN, FN were calculated from these numbers. A discrepancy was noted for phenotypic kanamycin resistance: the proportion of resistant strains is reported to amount to 37.3%, whereas the number of resistant strains and the number of total strains are both reported to be 118. |
| **Daniyarov 2023** | TP, FN, FP, TN were derived from Table 4 and Table 1. |
| **Deelder 2022** | TP, FN, FP, TN were calculated from sensitivity, specificity and number of resistant and susceptible strains. |
| **Gröschel 2021** | Sensitivity and specificity and total resistant and susceptible strains were used to calculate TP, FP, FN and TN. No decimals were reported for sensitivity and specificity. Therefore, these numbers were approximations. |
| **Guimarães 2020** | TP, FN, FP, TN were copied from Table II. Discrepancies were noted between deduced sensitivity and specificity and reported sensitivity and specificity in Supplementary Data Table III for KvarQ and CASTB and rifampicin resistance, and KvarQ and streptomycin resistance. The number of phenotypically resistant pyrazinamide strains reported for CASTB (TP + FN, four strains) did not match the number reported for other tools (six strains). |
| **Hall & Lima 2023** | TP, FN, FP, TN for Mykrobe were derived from Table S2 and Table S3, representing Illumina- and Nanopore-sequenced genomes, respectively. Strain numbers from both sources were summed. |
| **Hall & Rabodoarivelo 2023** | FN and FP for Illumina-sequenced strains were extracted from Table S2. TP and TN were calculated using the total number of total resistant and susceptible strains. |
| **Hasan 2023** | TP, FN, FP, TN were derived from Figure 1. |
| **He 2024** | TP, FN, FP, TN were extracted from Table 1. |
| **Hunt 2019** | TP, FN, FP, TN were copied from supplementary file 'accuracy_stats.tsv' for the Prospective dataset and using Mykrobe.CP3, except for rifabutin, PAS, cycloserine and linezolid, that were only reported for the Global Dataset. |
| **Iwamoto 2019** | TP, FN, FP, TN were copied from Table 5: predictions based on the standard library were used. |
| **Johnsen 2019** | TP, FN, FP, TN were calculated from Table 8 for Pointfinder and from Table 9 for PointFinder and PhyResSE. |
| **Kardan-Yamchi 2020** | TP, FN, FP, TN were calculated from sensitivity and specificity, as found in Table 6 and the proportion of phenotypically resistant strains reported in the Section 3.2. |
| **Lim 2023** | TP, FN, FP, TN were derived from Supplementary material 2. |
| **Liu 2024** | TP, FN, FP, TN were calculated from Table S3. |
| **Mahé 2019** | TP, FN, FP, TN were calculated from sensitivity and specificity data found in Supplementary Table S1 (data with default minimum threshold settings used per tool) and the proportion of phenotypically resistant strains reported in the Table 1. |
| **Morey-Léon 2023** | TP, FN, FP, TN were calculated in Table S2. |
| **Peker 2021** | TP, FN, FP, TN were calculated from Table S5 from TBProfiler and Mykrobe Illumina data. |
| **Phelan 2016** | TP, FN, FP, TN were counted in Table 1. |
| **Phelan 2019** | TP, FN, FP, TN data were calculated from sensitivity and specificity and total number of susceptible and resistant strains, as reported in Additional File 2, Table S7. Given the limited number of decimals reported for sensitivity and specificity, calculated numbers were approximations. Performance data for ReSeqTB were not included, since this concerned solely the use of the mutation library of ReSeqTB (run in TBProfiler) and not its variant calling pipeline. |
| **Qu 2024** | TP, FN, FP, TN were extracted from Table 4. |
| **Quagliaro 2023** | TP, FN, FP, TN were extracted from Tables 2 and 3. |
| **Rukmana 2024** | TP, FN, FP, TN were derived from Table 1. |
| **Schleusener 2017** | TP, FN, FP, TN were copied from Supplementary Dataset S4. |
| **Sharma 2024** | Uncorrected Mykrobe values for TP, FN, FP, TN were extracted from Table 1. |
| **Silcocks 2023** | TP, FN, FP, TN were calculated from sensitivity and specificity data from Figure 1 and total resistant and susceptible strains in Table 2. |
| **Vallejos-Sanchez 2025** | TP, FN, FP, TN were calculated from Table 1. |
| **Van Beek 2019** | TP, FN, FP, TN data were calculated from sensitivity and specificity and total number of susceptible and resistant strains as reported in Table 1. Given the limited number of decimals reported for sensitivity and specificity, these were approximations. |
| **Wang & Dong 2025** | TP, FN, FP, TN were extracted from Table 2. |
| **Wang & Lai 2025** | TP, FN, FP, TN were derived from Figure 1. |
| **Wang & Yu 2023** | TP, FN, FP, TN were extracted from Table 5. |
| **Wu 2020** | TP, FN, FP, TN were extracted from Table 3. |
| **Wu 2022** | TP, FN, FP, TN were extracted from Table 3. |
| **Yang 2022** | TP, FN, FP, TN were extracted from Supplementary Table 14. |

# Supplementary Table S2. Extracted quality control data for Domain 1

**Quality control data extraction table for Domain 1 (strain selection). Interpretation of bias is color coded as red (high risk of bias), grey (unclear risk of bias) or green (low risk of bias).**

As specified in Supplementary File S2, bias was assessed using three signalling questions:

1) A risk of sampling bias due to use of phylogenetically monotonous strains
2) A risk of sampling bias inherent to study design (a case control versus prospective study design)
3) A risk of exclusion bias due to inappropriate sample exclusions

|  | **Domain 1: Strain selection** | | |
| --- | --- | --- | --- |
|  | **Bias by phylogenetic diversity** | **Bias by study design** | **Bias due to inappropriate exclusions** |
| **Bradley 2015** | - Validation set of 1,609 strains taken from a composite dataset of Walker et al. 2015 (57). These strains were original (not yet published) at the time of this study (2014-2015). - The distribution of lineages was not reported. - Rifampicin-resistance in 303/1,609 strains (18.8%).   The authors report that strains included represented all seven global clades. However, numbers on the lineage distribution are not provided. Risk was rated unclear. | No details were given on how strains were selected for inclusion. Risk was rated unclear. | No explicit mention of sample exclusion. The source dataset(57) was only partially included, with no reasons specified on strain exclusions. Risk of bias was rated unclear. |
| **Chatterjee 2017** | - Validation set of 74 original strains were randomly included from two strain collections in Mumbai, India - Lineage diversity: 27/74 (37%) were Beijing/East Asia (Lineage 2), 25/74 (34%) had belonged to the Delhi/Central Asia lineage (Lineage 3), 17/74 (23%) were European/American (Lineage 4) and 5/74 (7%) were East Africa/Indian Ocean (Lineage 1). - Resistance distribution: 35/74 (47%) were pan-sensitive, 16/74 (22%) were MDR, 13/74 (18%) were pre-XDR, 1/74 (1%) was XDR.   This dataset contained phylogenetically heterogeneous strains. Risk of bias was rated low. | Strains were randomly selected from two strain collections. Risk was rated low. | No mention of sample exclusions in randomly collated set. Risk was rated low. |
| **Che 2022** | - 59 strains rifampicin-resistant strains were randomly selected among stocked isolates. These were collected between 1 January 2018 and 30 December 2019 (exclusion criteria based on age and comorbidities were used) in Ningbo, China. - 53/59 belonged to lineage 2 (89.8%), 6/59 (10.2%) belonged to lineage 4. - Rifampicin-resistance rate is 100%, 49/59 were MDR-TB (83.1%), 16/59 were pre-XDR-TB (27.1%), 7/59 were XDR-TB (11.9%).   This is a medium-size dataset of strains almost exclusively belonging to lineage 2. Risk of bias was rated high. | This study only included 59 rifampicin-resistant strains (out of 704 prospective strains) and excluded patients with positive HIV status, pregnant women, age below 18 years and renal or liver disease. Risk of bias was rated high. | No sample exclusions or failed analyses were mentioned in a randomly collated set. Risk was rated low. |
| **Chen 2025** | - A total of 36,385 strains from 45 countries across six continents were selected from public datasets. The number of strains varied per drug studied. - The lineage distribution was as follows: 17,134 L4 strains (47.1%), 9695 L2 strains (26.6%), 5438 L3 strains (14.9%), 3434 L1 strains (9.4%), 63 L5 strains (0.17%), 59 L6 strains (0.16%), 3 L7 strains (0.0082%), 559 animal-associated strains (1.5%) comprising *M. bovis-La1* (426), *M. orygis-La3* (121), and *M. caprae-La2* (12). - Rifampicin-resistance in approximately 28% of strains.   This is a heterogeneous and large dataset. Risk of bias was rated low. | This was a dataset compiled from multiple sources. No details were given on how strains were selected for inclusion. Risk was rated unclear. | No mention of strain exclusions in a non-randomly collated dataset. Risk was rated unclear. |
| **Cloutier Charette 2024** | - Validation strains concerned 32 original Madagascan MDR-TB strains isolated between 2010 and 2017, and 40 matched DS-TB isolates. - Lineage distribution not reported for the full strain set. - Resistance distribution: 32 strains were MDR, and 40 were susceptible isolates   Phylogenetic distribution of included strains not mentioned in medium-sized dataset. Risk of bias was rated unclear. | Strains were sampled in a case-control fashion. Risk of bias was rated high. | No mention of strain exclusions in a non-randomly sampled dataset.  Risk was rated unclear. |
| **Coll 2015** | - Validation set of 792 original strains from geographically diverse origin (including China, Pakistan, Malawi, Portugal, Russia, Canada) (see publication’s Supplementary Table 3). - Lineage diversity: Lineage 1 (East African Indian spoligotype family: 68, 8.6%), Lineage 2 (Beijing spoligotype: 182, 23%), Lineage 3 (Central Asian: 86, 10.9%) and Lineage 4 (456 isolates, 57.5%). - Resistance distribution: 365/792 (46%) resistant to at least one drug, 262/792 (33%) MDR-TB, 54/792 (6.8%) XDR-TB.   Over half of included strains belonged to lineage 4. Risk of bias was rated high. | No details were given on how strains were selected for inclusion. Risk was rated unclear. | Only subsets of genomes of source datasets were used for in this dataset (for example, among others, 21 strains of PRJEB2138, which contains 1001 strains). Risk of bias was rated unclear. |
| **Daniyarov 2023** | - Ten original strains processed by the Kazakthstan national reference center. - All ten strains were lineage 2, belonging to two separate outbreaks - All ten strains had a pre-XDR phenotype.   This concerned a small and homogeneous dataset. Risk of bias was rated high. | This concerned ten pre-XDR-strains. Risk of spectrum bias was rated high. | No mention of strain exclusions in a non-randomly collated dataset.  Risk was rated unclear. |
| **Deelder 2022** | - Strains were selected per tuberculostatic from a 32,689 strain composite dataset of public domain strains: isoniazid (n = 1835), rifampicin (n = 2045), ethambutol (n = 1999), para-aminosalicylic acid (n = 1114), cycloserine (n = 833), ethionamide (n = 2118). - Lineage distribution in 32,689 strains: lineages 1 (9.6%), 2 (25.2%), 3 (11.4%) and 4 (51.0%). - The rate of rifampicin resistance was 8.1%.   Over half of included strains belonged to lineage 4. Risk of bias was rated high. | Strains were included when second-line phenotypic DST results were available. This may confer bias toward inclusion of strains with more resistant or complicated resistance profiles. Risk was rated unclear. | No mention of sample exclusion or failed analyses in a non-randomly collated set. Risk was rated unclear. |
| **Gröschel 2021** | - A sample of 20,408 isolates was collated from public datasets, of which 19,880 strains with high-quality sequences were retained. - Phylogenetic diversity: lineage 4: 52%, lineage 2: 21%, lineage 3: 15%, lineage 1: 10%, *M. bovis*: 0.6%, lineage 6: 0.3%, and lineage 5: 0.2%. - 13,817 of 20,408 (67.7%) of strains were phenotypically susceptible to first-line drugs, 4743 (23.3%) were phenotypically MDR, and 396 (1.9%) were phenotypically XDR.   Over half of included strains belonged to lineage 4. Risk of bias was rated high. | This was a dataset compiled from multiple sources. No details were given on how strains were selected for inclusion. Risk was rated unclear. | No mention of sample exclusion or failed analyses after selection of high-quality NGS files. The validation dataset was compiled from public datasets. At least several datasets were incompletely included (for example, 848 strains of 1001 for PRJEB2138).  Risk was rated unclear. |
| **Guimarães 2020** | - Dataset of 71 original strains from Pará, Brazil, of which part was published earlier.(58) - Only lineage 1 strains were included. - 16/71 (22.5%) were MDR, 56/71 (78.9%) were non-MDR strains. (Note: in Table II in this publication 15 reference MDR strains are reported and 56 non-MDR strains.)   This concerns a set of strains exclusively belonging to lineage 1. Risk of bias was rated high. | No details were given on how strains were selected for inclusion. Risk was rated unclear. | All 71 strains were analysed for all tools, except for two pyrazinamide-resistant strains not analysed for CASTB. Reasons were not indicated. Risk of bias was rated high. |
| **Hall & Lima 2023** | - A validation set of 44, 709 public Illumina-sequence strains and 138 Nanopore-sequenced strains. - The lineage distribution is as follows: **Lineage** \| **Illumina** \| **%** \| **Nanopore** \| **%**; La1 \| 239 \| 0.53% \| 0 \| 0%; La2 \| 7 \| 0.02% \| 0 \| 0%; La3 \| 71 \| 0.16% \| 0 \| 0%; L1 \| 3907 \| 8.74% \| 32 \| 13.45%; L2 \| 12870 \| 28.79% \| 38 \| 15.97%; L3 \| 5803 \| 12.98% \| 9 \| 3.78%; L4 \| 20731 \| 46.37% \| 59 \| 24.79%; L5 \| 63 \| 0.14% \| 0 \| 0%; L6 \| 78 \| 0.17% \| 0 \| 0%; L7 \| 3 \| 0.01% \| 0 \| 0%; L9 \| 1 \| 0.002% \| 0 \| 0% - Of 40,548 strains with rifampicin results, 14731 are resistant (36.3%) This is a large and heterogeneous dataset. Risk of bias was rated low. | The strain sampling procedure was not specified.  Risk of bias was rated unclear. | No mention of strain exclusions, except those with insufficient sequencing quality, in a non-randomly collated dataset.  Risk was rated unclear. |
| **Hall & Rabodoarivelo 2023** | - Ninety-one isolates from Madagascar (consecutive, phenotypically confirmed MDR isolates and geographically matched sensitive isolates) and 41 isolates from South Africa (selection criterium: isolates with available WGS data). Nineteen additional isolates from England were not included in the study of genotypic-phenotypic resistance prediction. - Distribution of lineages not reported. - Rifampicin resistance in 79/151 (52.3%) of isolates. Rate of XDR not reported.   This is a medium-size dataset with no information on phylogenetic diversity. Risk of bias was rated unclear. | Ninety-one of 132 strains were selected in a case-control fashion. Risk of bias was scored high. | Fifty-one samples of an initial 208 isolates were excluded due to insufficient sequencing depth (n = 44), non-matched Illumina and Nanopore data (n = 1), and (likely) impure culture (n = 12). As these variables are unlikely to introduce bias, risk for exclusion bias was rated low. |
| **Hasan 2023** | - 1526 strains, susceptible to first-line tuberculostatics were downloaded from the curated ReSeqTB database to study discrepancies between WGS-based and phenotypic DST. - Lineage distribution was not reported. - All strains were susceptible to first-line drugs.   Phylogenetic diversity could not be assessed as lineage distribution was not reported. Risk was rated unclear. | This concerned exclusively susceptible strains. Risk of spectrum bias was rated high. | No mention of strain exclusions in a non-randomly collated dataset.  Risk was rated unclear. |
| **He 2024** | - 110 strains, randomly selected from 6000 clinical samples isolated in Zhejiang, China, previously published in 2019 (59) - Eighty-seven of 110 strains belonged to lineage 2; 23 of 110 belonged to lineage 4 (from (60)). - Multi-drug resistance in 100 of 110 strains.   This is a medium-size dataset exclusively consisting of lineage 4 strains. Risk of bias was rated high. | Strains were sampled in a case-control fashion. Risk of bias was rated high. | No mention of strain exclusions in a non-randomly collated dataset. Risk was rated unclear. |
| **Hunt 2019** | - Validation set consisted of 4,362 prospective strains from the CRyPTIC database (from Italy, Germany, the Netherlands and the UK).(51) - The distribution of lineages was not reported and could only be derived from raw data in the source publication.(51) - Distribution of drug-resistance phenotypes not reported. Rifampicin-resistance in extracted data in 133/4,362 strains (3.0%).   The lineage distribution was not reported. Risk was rated unclear. | This concerned an international prospective strain collection. Risk was rated low. | No mention of sample exclusion or failed analyses in a prospective dataset. Risk was rated low. |
| **Iwamoto 2019** | - Validation set consisted of 191 original strains extracted from two Japanese collections. - No information on lineage diversity was reported. - Multi-drug-resistant phenotype (MDR) in 165 of 195 strains (84.6%).   This is a dataset with no information on phylogenetic diversity. Risk of bias was rated unclear. | WGS was performed on single colonies from stock cultures to avoid hetero-resistant phenotypes. This might increase the risk of spectrum bias. | No mention of sample exclusion or failed analyses in a non-randomly selected dataset. Risk for exclusion bias was rated unclear. |
| **Johnsen 2019** | - Validation strains: 2,480 isolates selected from previously published strain sets of 6,465 strains (61) and 91 isolates (5) - The lineage distribution was not reported for the strain selection used by the authors. - Rifampicin resistance in 24.3% of both validation sets.   Lineage distribution was not reported. Risk of bias was unclear. | The sampling procedure was not specified. Risk of bias was rated unclear. | No mention of strain exclusions in a non-randomly collated dataset.  Risk was rated unclear. |
| **Kardan-Yamchi 2020** | - A dataset of 35 original strains collected in Tehran, Iran. - Phylogenetic diversity: 1/35 Lineage 1 (2.9%), 15/35 lineage 4 (42.9%), 7/35 lineage 3 (20%), 12/35 lineage 2 (34.3%) - These concerned 35 RR-/MDR-strains, of which 13 pre-XDR or XDR   This is a phylogenetically diverse dataset. Concern for bias was rated low. | Out of 845 positive cultures, 37 rifampicin-resistant isolates were selected. Inclusion criteria not further specified.  Risk was rated unclear. | Two strains with ambiguous phenotypic results were excluded. This increases the risk of spectrum bias. Risk for exclusion bias was rated high. |
| **Lim 2023** | - A validation set of original 3,808 isolates collected as part of a national surveillance program in Singapore from 2019 to 2022. - 1847 samples (48 %) belonged to East-Asian lineage 2; 1334 samples (35%) belonged to lineage 1, and 535 samples (14 %) belonged to lineage 4 - Rifampicin-resistance in 2.1% (62 out of 2996)   A large dataset with three major lineages well represented and L2 predominating. Risk was rated low. | Samples from 2019-2020 (938 strains) were selected for multidrug resistance. Samples from 2021 were included prospectively.  Risk was rated unclear. | No mention of strain exclusions in a non-randomly collated dataset.  Risk was rated unclear. |
| **Liu 2024** | - A validation set of 297 MDR cases isolated by the Taiwan Centers for Disease Control (Taiwan CDC) between 2019 and 2022. - 188 out of 297 strains belonged to lineage 2 (63.3%), 29 strains belonged to lineage 1 (9.8%) and 80 belonged to lineage 4 (27.0%) - All strains were multi-drug resistant.   This concerned a dataset of mostly lineage 2 strains. Risk was rated high. | This concerned a dataset of only MDR strains. Risk of spectrum bias was rated high. | No mention of strain exclusions in a non-randomly collated dataset.  Risk was rated unclear. |
| **Mahé 2019** | - Validation set contained 6,574 genomes, gathered from several studies (Coll et al. 2018, Bradley et al. 2015, Schleusener et al. 2017 and the ReSeqTB platform). (For full citations, see Mahé et al. 2019.)(4) - Lineage distribution: lineage 1 (East-African/Indian Ocean): 9.5%, lineage 2 (Beijing/East-Asia): 15%, lineage 3 (Delhi/Central-Asia): 16%, and lineage 4 (European/American): 59%; 0.5% other lineages. - Distribution of drug resistance phenotypes not reported. Rifampicin-resistance in extracted data in 1,237/6,574 (18.8%).   Over half of included strains belonged to lineage 4. Risk of bias was rated high. | This was a dataset compiled from multiple sources. No details were given on how strains were selected for inclusion. Risk was rated unclear. | This concerned a composite dataset. Apart from deduplication, no mention of strain exclusions.  TBProfiler and Mykrobe did not provide results for three and two samples, respectively. Risk for exclusion bias was rated high. |
| **Morey-Léon 2023** | - Eighty-eight multi-drug-resistant isolates from Ecuador (selection criterium: ‘convenience sampling’) - All strains concerned lineage 4. - Multi-drug resistance in 52 out of 88 strains; pre-XDR in 10 out of 88 strains.   This is a medium-size dataset exclusively consisting of lineage 4 strains. Risk of bias was rated high. | Strains were sampled for ‘convenience’.  Risk of bias was scored unclear. | No mention of strain exclusions in a non-randomly collated dataset. Risk was rated unclear. |
| **Peker 2021** | - A study of 24 strains collected in Germany and the Netherlands for comparing Illumina and Nanopore sequencing. Of these, nine isolates were used for determining QC parameters. - Ten of 12 (83.3%) strains are L2 (lineage 2.2.1). Two strains are lineage 4 (4.8 and 4.3.3). - Seven strains were multi-drug-resistant 58.3%) and five had a pre-XDR phenotype (41.7%).   This small dataset consisted mostly of L2 strains. Risk was rated high. | The 12 validation strains were selected for their resistant phenotype. Risk of bias rated high. | No mention of strain exclusions in a non-randomly collated dataset.  Risk was rated unclear. |
| **Phelan 2016** | - Validation set of 10 original strains isolated in Lisbon + H37Rv - All strains belonging to lineage 4. - Resistance distribution: 7/10 XDR, 3/10 MDR, H37Rv is pan-susceptible   This is a small dataset of strains belonging to lineage 4. Risk of bias was rated high. | No details were given on how strains were selected for inclusion. Risk was rated unclear. | No mention of sample exclusions in non-randomly assembled dataset. Risk was rated unclear. |
| **Phelan 2019** | - A validation dataset of 17,239 strains from public online sources. Three strains used for ONT sequencing. - Distribution of lineages: lineage 1: 1,885/17,239 (10.9%), lineage 2: 3721/17,239 (21.6%), lineage 3: 2886/17,239 (16.7%), lineage 4: 8531/17,239 (49.5%), lineage 5: 35/17,239 (0.2%), lineage 6: 37/17,239 (0.2%), lineage 7: 1/17,239 (0.0%), *M. bovis* 129/17,239 (0.7%). - Distribution of resistance phenotypes: MDR: 3845/17,239 (22.3%), XDR: 345/17,239 (2.0%).   This is a heterogeneous and large dataset. Risk of bias was rated low. | This was a dataset compiled from multiple sources. No details were given on how strains were selected for inclusion. Risk was rated unclear. | There is no mention of sample exclusion or failed analyses. For several source datasets, only subsets were included (for example, 47 of 92 strains for PRJEB7727, and 37 of 1001 strains for PRJEB2138).  Risk of bias was rated unclear. |
| **Qu 2024** | - Out of a collection 115 clinical isolates collected between 2020 and 2021 in Guangdong, China, 82 strains were selected. - Lineage distribution was not reported. - Resistance distribution: rifampicin resistance in 62 out of 82 isolates (75.6%).   Phylogenetic distribution of included strains not mentioned in medium-sized dataset. Risk of bias was rated unclear. | The strain sampling procedure was not specified.  Risk of bias was rated unclear. | No mention of strain exclusions in a non-randomly collated dataset. Risk was rated unclear. |
| **Quagliaro 2023** | - 257 validation strains were randomly selected among stocked isolates from 2015 to 2021, isolated in France - Phylogenetic diversity (as determined by PhyResSE): lineage 4: 155/227 (68.2%), lineage 3: 13/227 (5.7%), lineage 1: 31/227 (13.7%), lineage 2: 12/227 (5.3%), lineage 6: 6/227 (2.6%), Mycobacterium bovis: 6/227 (2.6%), lineage 5: 4/227 (1.8%). - Multi-drug resistance in 5 of 222 strains (2.3%).   Over half of included strains belonged to lineage 4. Risk of bias was rated high. | Strains were randomly selected from a strain collection. Risk was rated low. | Except for exclusion of 30 strains due to inadequate sequencing quality, no sample exclusions or failed analyses were mentioned. Risk was rated low. |
| **Rukmana 2024** | - Thirty-four original Mtb isolates were tested for pyrazinamide resistance, isolated in Indonesia. - The lineage distribution was as follows: Beijing (lineage 2) in 21/34 (61.7%), Harlem and Lam (lineage 4) in 11/34 (32.4%), Indo-oceanic (lineage 1) in 2/34 (5.9%) - Rifampicin-resistance not reported. Pyrazinamide resistance in 8 of 34 isolates (23.5%)   This concerned a small dataset consisting mostly of lineage 2 strains. Risk was rated high. | Sampling procedure was not specified. Risk was rated unclear. | No mention of strain exclusions in a non-randomly collated dataset.  Risk was rated unclear. |
| **Schleusener 2017** | - Validation set of 91 strains from Sierra Leone - Calculated lineage distribution as follows: lineage 1: 4/92 (4.3%), lineage 2: 4/92 (4.3%), lineage 5: 6/92 (6.5%), lineage 6: 14/92 (15.2%), lineage 4: 64/92 = 69.6%. Note: the number of strains in Table 3 (that contains phylogenetic data) adds up to 92 strains, and not 91 strains. - Resistance distribution: 10/91 were MDR (11%) (as reported in Supplementary Data set S1).   Over half of included strains belonged to lineage 4. Risk of bias was rated high. | No details were given on how strains were selected for inclusion in the dataset. Risk was rated unclear. | No mention of sample exclusion or failed analyses. Source dataset, consisting of 97 strains, was incompletely included.(62) Risk was rated unclear. |
| **Sharma 2024** | - 1510 stocked isolates from the Canadian National Microbiology Laboratory, sequenced between 2018 to 2022 - The phylogenetic distribution is not reported. - Drug resistance distribution: 90.1% of strains were susceptible, multi-drug resistance in 1%, isoniazid mono-resistance in 6.6%, rifampicin-mono-resistance in 0.3% and pyrazinamide-mono-resistance in 1.6%.   This concerns a retrospective original dataset of which the phylogenetic distribution was not reported.  Risk was rated unclear. | Inclusion based on availability of WGS and pDST, without further inclusion criteria specified.  Risk was rated unclear. | No mention of strain exclusions in a non-randomly collated dataset.  Risk was rated unclear. |
| **Silcocks 2023** | - This concerned 2,619 isolates collected in clinical studies, published since 2016, collected in Ho Chi Minh City, Vietnam. - The lineage distribution was as follows: lineage 2: 1,615, (61.7%); lineage 1: 649 (24.8%); lineage 4: 275 (10.5%); lineage 3: 2 strains (0.08%). - Multi-drug resistance in 69 out of 1786 strains (3.9%)   This concerned a dataset of predominantly lineage 2 strains. Risk of bias was rated high. | Strains were collected as part of various clinical studies and filtered based on age and HIV-status.  Risk was rated unclear. | No mention of strain exclusions in a non-randomly collated dataset.  Risk was rated unclear. |
| **Vallejos-Sanchez 2025** | - Retrospective analysis of 2,917 Peruvian clinical isolates, including 14.6% mixed infections, published since 2016. - Lineage diversity not reported for the full strain set - Rifampicin resistance in 615 of 2917 strains (21.9%)   This is a large dataset with no information on phylogenetic diversity. Risk of bias was rated unclear. | The strain sampling procedure was not specified.  Risk of bias was rated unclear. | No mention of strain exclusions in a non-randomly collated dataset.  Risk was rated unclear. |
| **Van Beek 2019** | - Validation set consisted of 211 of 213 strains isolated strains in Finland in 2014. - No information on lineage diversity was reported. - Multidrug resistant strains: 8/211 = 3.8%.   This is a medium-size dataset with a low resistance prevalence and no information on phylogenetic diversity. Risk of bias was rated unclear. | This is a dataset of consecutive strains. Two strains of this consecutive series were not available for inclusion in this study (reasons not clarified). Risk was rated unclear. | Analysis of three isolates failed (one for TBProfiler and two for both TGS-TB and KvarQ). Risk of bias was rated high. |
| **Wang & Dong 2025** | - Validation set of 209 resistant strains from Hainan Island, China, collected between 2019 and 2021, but not made public before. - Lineage diversity: 6 strains (2.9%) lineage 1, 174 strains lineage 2 (83.3%), and 29 strains (13.9%) lineage 4. - Resistance distribution: all strains were MDR-TB (100%).   Over half of included strains belonged to lineage 2 (84.3%). Risk of bias was rated high. | Strains were sampled in a case-control fashion. Risk of bias was rated high. | The 209 isolates were selected after exclusion of strains with discordant phenotypic results. Risk of bias was rated high. |
| **Wang & Lai 2025** | - Validation set of 113 strains selected out of 138 Mtb strains collected in Ganzhou, China - Lineage diversity not reported - Rifampicin resistance in 29 out of 113 strains (25.7%)   This is a medium-size dataset with no information on phylogenetic diversity. Risk of bias was rated unclear. | The strain sampling procedure was not specified.  Risk of bias was rated unclear. | 25 out of 138 initially collected strains were excluded due to technical variables (failure to re-culture, failed DNA extraction or sequencing quality control). Strain exclusions in a non-randomly collated dataset: bias was rated unclear. |
| **Wang & Yu 2023** | - Validation set of 202 resistant strains from Hainan Island, China, collected between 2019 and 2021 (a similar set as the 209 strains analysed by Wang & Dong 2025) - Lineage diversity: 6 strains (2.9%) lineage 1, 169 strains lineage 2 (83.7%), and 27 strains (13.4%) lineage 4. - Resistance distribution: all strains were MDR-TB (100%).   Over half of included strains belonged to lineage 2 (83.7%). Risk of bias was rated high. | Strains were sampled in a case-control fashion. Risk of bias was rated high. | Strain exclusions were due to technical reasons (failure to re-culture, contamination, failed sequencing). Contrary to Wang & Dong 2025, no exclusions based on phenotypic DST discordances mentioned.  No mention of strain exclusions in a non-randomly collated dataset.  Risk was rated unclear. |
| **Wu 2020** | - Validation set of 306 original, randomly selected strains from a strain collection of a Shanghai hospital. - 291/306 (95.1%) strains belonged to lineage 2, 15/306 (4.9%) belonged to lineage 4. - 276 RR-TB (90.2%) and 30 (9.8%) rifampicin-sensitive clinical isolates.   This concerns a dataset of strains almost exclusively belonging to lineage 2. Risk of bias was rated high. | Strains were randomly selected from a strain collection. Risk was rated low. | No mention of sample exclusion or failed analyses in a randomly collated dataset. Risk was rated low. |
| **Wu 2022** | - A dataset of 182 clinical isolates from a Shanghai hospital collected prospectively between 1 August 2019 and 30 October 2020. Selection criteria not mentioned. - Phylogenetic diversity: lineage 2: 164/182 (90.1%); lineage 4: 13/182 (7.1%); mixed lineage 2-4 call: 4/182 (2.2%), *Mycobacterium bovis*: 1/182 (0.1%). - The MDR-rate is 59/182 (32.4%).   This concerns a dataset of strains almost exclusively belonging to lineage 2. Risk of bias was rated high. | No details were given on how strains were selected for inclusion in the dataset. Risk was rated unclear. | Among 211 initial isolates, six strains (6/188 = 3.2%) were excluded due to poor growth for phenotypic DST. This might confer a risk of spectrum bias. Risk was rated high. |
| **Yang 2022** | - A total of 3,177 strains were selected from public datasets. The number of strains varied per drug studied. - Lineage distribution of drug-resistance prediction strain set was not reported. - Multi-drug resistance in 1470 of 3177 strains (46.3%).   No information on phylogenetic distribution. Risk of bias was rated unclear. | No details were given on how strains were selected for inclusion. Risk was rated unclear. | No sample exclusions or failed analyses were mentioned in a non-randomly collated dataset. Risk of bias was rated unclear. |

# Supplementary Table S3. Extracted quality control data for Domain 2

**Quality control data extraction table for Domain 2 (the index test, whole-genome sequencing). Interpretation of bias is color coded as red (high risk of bias), grey (unclear risk of bias) or green (low risk of bias).**

As specified in Supplementary File S2, bias was assessed using two signalling questions:

1) Were the training strains different from the validation strains?
2) What is the risk of measurement bias due to 1) variable tool’s settings, 2) variable tool’s versions, 3) variables in whole-genome sequencing?

|  | **Domain 2: the index test** | | | | |
| --- | --- | --- | --- | --- | --- |
|  | **Were the training strains different from the validation strains?** | **Measurement bias due to variable tool’s settings** | **Measurement bias due to variable tool’s versions** | | **Measurement bias due to variables in whole-genome sequencing** |
| **Bradley 2015** | 1,920 isolates were used for training Mykrobe and 1,609 different isolates were used for validation (57). Risk was rated low. | Default settings (first version) | Mykrobe: first version  KvarQ: version not mentioned. | | All WGS analyses were performed in reference laboratories; low concern that inappropriate variables in test technology have affected prediction performance. Mean depth was not reported. Required read depth for variants was 5x. Source publication mentions required coverage of included strains of ≥88%.(57)  Low concern for bias. |
| **Chatterjee 2017** | These concerned original strains submitted online Sep 25^th^ 2017 (accession SRP101835), unlikely to be included in training data of Mykrobe Predictor version 0.4.2. Risk was rated low. | Default settings (assumed) | Mykrobe Predictor version 0.4.2 | | WGS was performed at an internationally accredited laboratory. Risk for bias considered low. The average variant depth for each isolate was ≥ 10 reads. Genome coverage was not reported. |
| **Che 2022** | This concerned an original dataset not likely to have been used to train any of the evaluated tools (deposited as PRJNA862152 on 2022-10-07). Risk was rated low. | Default settings (assumed) | TBProfiler: webserver; version or date not mentioned. | | Illumina sequencing according to manufacturer’s instructions. Unclear reporting of depth and coverage. Risk was rated unclear. |
| **Chen 2025** | The validation strains used here were the same set used for the composition of the 2021 WHO catalogue of Mtb resistance-associated variants,(44) which serves as the basis for the TBProfiler catalogue (cfr. https://github.com/jodyphelan/tbdb/blob/master/mutations.csv) and the WHO catalogues.  Risk was rated high. | All tools were used with default settings (assumed). | TBProfiler v5.0.0  GenTB v1  MD-CNN v1.0  SAM-TB v1  WHO 2023 Catalogue | | This concerns a composite dataset of diverse studies. Depth and coverage requirements were not mentioned. Risk was rated unclear. |
| **Cloutier Charette 2024** | This concerned a private dataset, not mentioned to have been deposited publicly.  Risk was rated low. | Mykrobe was used with default settings (assumed) | Mykrobe v0.12.1 | | Illumina sequencing at the Wellcome Center for Human Genetics. The average depth of included samples was 30x. Required genome coverage was not reported.  Risk was rated unclear. |
| **Coll 2015** | TBProfiler’s mutation library was created from variants in two publicly available databases (TBDReaMDB and MUBII-TB-DB) and a literature review, yielding 34 studies that reported resistance-associated mutations. These were different from the publications used in the validation study. Risk was rated low. | Default settings (first version) | TBProfiler: first version | | The authors indicate that ‘all collections had Illumina raw sequencing data (minimum read length 50 bp) and drug susceptibility data from recognised testing protocols’. Samples had ‘depth of coverage ranging from 20x to 1000x’. Genome coverage not unambiguously reported.  Risk for bias was rated unclear. |
| **Daniyarov 2023** | Strains were deposited as PRJNA481625 on 2022-11-20.  Risk was rated unclear. | All tools were used with default settings (assumed). | Versions not mentioned for TBProfiler, Mykrobe, CASTB or ResFinder | | Strains were mapped with an average read depth of 160x and mean genome coverage of 98.95%.  Risk was rated low. |
| **Deelder 2022** | No project accession identification of source strains or references to source datasets mentioned.(63) Therefore, unclear whether isolates had been used in earlier training or validation cohorts. | Default settings (assumed) | TBProfiler: version not mentioned. | | This concerns a composite dataset of diverse studies. Depth and coverage requirements were not mentioned. Risk was rated unclear. |
| **Gröschel 2021** | Validation strains were drawn from datasets previously used to train tools, including those described by Bradley et al. 2015 (used to train Mykrobe v.0.7.0)(1,9) and Coll et al. 2018 (PRJEB10385; used to train TBProfiler v.2.0).(3,61) Risk of bias was rated high. | Default settings (assumed) | GenTB: first version  TBProfiler webserver version 3.0.0  Mykrobe desktop version 0.90 | | This concerns a composite dataset of public domain strains. Only strains with a 10x depth across > 95% of the genome were studied.  Risk was rated low. |
| **Guimarães 2020** | Strains were public as PRJNA494931 from 2018-11-01. Possible use of published strains for training of the used tools was unclear. | Default settings (assumed) | TBProfiler version 2.8.6  KvarQ version 0.12.2  PhyResSe version not mentioned  Mykrobe version 0.8.1  MTBseq version 1.0.4  CASTB version not mentioned  RSniffer version not mentioned | | Illumina sequencing. Strains were sequenced “with a coverage of 250x”. No mention of average depth or genome coverage. Risk was rated unclear. |
| **Hall & Lima 2023** | This concerned a study of public genomes of which the sample’s accessions were not specified.  Risk was rated unclear. | Mykrobe was used with default settings (mentioned) | Mykrobe v0.12.1 | | 44 709 and 138 publicly available Illumina and Nanopore samples. Samples with an average read depth <15x, or where more than 5 % of the reads mapped to contaminants were excluded. Achieved genome coverage not mentioned.  Risk was rated unclear. |
| **Hall & Rabodoarivelo 2023** | Madagascan and South African isolates used in the validation set of this study were original strains first submitted online 2022-03-03 (ENA accession PRJEB49093). It is unclear whether these had been used for training of Mykrobe version 0.10.0. | For Oxford Nanopore sequencing, non-default settings were used. Default Mykrobe settings assumed for Illumina-sequenced isolates. | Mykrobe version 0.10.0 | | Illumina sequencing performed according to manufacturer’s instructions. Isolates with decontaminated depth less than 30x (Nanopore) or 20x (Illumina) were not analysed. Requirements for genome coverage were not mentioned. Risk for bias was rated unclear. |
| **Hasan 2023** | No accession identifiers were provided.  Risk was rated unclear. | TBProfiler was used with default settings (assumed) | TBProfiler version not mentioned. | | Strains were downloaded from a curated, quality-controlled database, ReSeqTB. Required genome coverage an depth were not mentioned.  Risk was rated unclear. |
| **He 2024** | This is a study testing original strains that were publicly deposited five years earlier.(60) It is unclear whether these strains had been used in any of the tool’s training set. | All tools were used with default settings (assumed). | TB Profiler v3.0.7  GenTB v7.24.0  PhyResSE v1.0 Mykrobe v0.12.1  SAM-TB: version not mentioned | | Illumina sequencing performed according to manufacturer’s instructions. Sequencing depth reported as ‘depths of at least 50-fold coverage’. Genome coverage not mentioned. Risk of bias considered unclear. |
| **Hunt 2019** | Tools were trained with other strains than those used in this novel prospective validation set (first described in 2018).(51) Risk was rated low. | Default settings (mentioned) | KvarQ commit d693f561d205c9a3f9b9c705e2  fefecdeb715cc8 (v.0.2)  MTBseq: version 1.0.3  TBProfiler commit 327e431c3e 9de2897a88  5fabe6bfede1421b2470 (v.0.3.9)  Mykrobe: 0.7.0 | | Sequencing was performed in experienced reference laboratories. Average sequencing depth and genome coverage of included strains not mentioned, neither in the source publication.(51) Risk was rated unclear. |
| **Iwamoto 2019** | This concerned an original dataset not likely used to train any of the evaluated tools (submitted online 15-MAR-2019). Risk was rated low. | Both default settings and adapted method for variant calling are used (meta-analysis performed on data with default settings). | TBProfiler, online version, September 2018  TGS-TB version 2  PhyResSE version 1.0  CASTB version 1.5 | | Illumina sequencing according to manufacturer’s instructions. Sequencing depth and coverage not reported. Risk was rated unclear. |
| **Johnsen 2019** | Validation strains were selected from previously used validation sets (Coll et al. 2018, (61)) and Schleusener et al. 2019 (PRJEB7727; 91 strains from Sierra Leone, (5)). The strains from Coll et al. (61) were, as stated, also used for compiling the mutation library of ResFinder. The possible overlap of validation and training strains confers a risk of biased performance. | All tools were used with default settings (assumed). | Versions not mentioned for ResFinder and PhyResSE | | This concerns a composite dataset of diverse studies. Depth and coverage requirements were not mentioned. Risk was rated unclear. |
| **Kardan-Yamchi 2020** | This concerned an original dataset unlikely to have been used to train any of the evaluated tools (PRJNA566379 deposited 2019-09-20). Risk was rated low. | MTBseq was used in low variant frequency detection mode (at least 2-fold coverage and 5% allele frequency). Risk of bias was rated unclear. | MTBseq: version not mentioned | | Illumina sequencing performed in a supranational TB reference laboratory according to manufacturer’s instructions. All strains had a mean genomic coverage depth of 50x. Genome coverage not mentioned separately. Risk was rated unclear. |
| **Lim 2023** | This concerns a private dataset, not deposited publicly.  Risk was rated low. | TBProfiler was used with default settings (assumed) | TBProfiler version not mentioned. | | Illumina sequencing performed in a reference laboratory. Samples were only included if sequencing depth > 50x. Genome coverage not mentioned.  Risk was rated unclear. |
| **Liu 2024** | Strains were publicly deposited but no accession information was provided. Risk was rated unclear. | TBProfiler was used with default settings (assumed) | TBProfiler v.4.1.1 | | Illumina sequencing was performed according to manufacturer’s instructions. Coverage and depth not mentioned.  Risk was rated unclear. |
| **Mahé 2019** | The validation set was not independent of the set used to train the isolates: 1804 of 6574 isolates were used to train Mykrobe (see Supplementary data, file dataset.csv, label ‘mykrobe_derivation’). Risk was rated high. | Default settings (mentioned) | TBProfiler command-line version 0.3.4  Mykrobe v0.3.3-0-gc211bf2 | | This concerns a composite dataset of public domain strains. Sequencing depth and coverage not mentioned. Risk was rated unclear. |
| **Morey-Léon 2023** | This is a study using original strains, deposited as PRJNA827129 on 31-Oct-2022. Risk was rated low. | All tools were used with default settings (mentioned). | TB-Profiler v5.0, PhyResSE v1.0, Mykrobe v0.12.2 [sic], KvarQ v0.12.2 [sic],  SAM-TB: version not mentioned | | Illumina sequencing performed according to manufacturer’s instructions. Isolates with a sequencing depth of < 20x or a coverage of < 90% were excluded. Low concern for bias. |
| **Peker 2021** | The sequencing data were deposited publicly on 2021-04-09 as PRJNA720906. These strains were not used in the training data of TBProfiler or Mykrobe. Risk was rated low. | Both tools were used with default settings (mentioned). | Mykrobe v0.8.0  TBProfiler v2.8.6 | | This concerned a comparison of Illumina and Nanopore sequenced Mtb strains. Illumina sequencing performed in a reference laboratory according to manufacturer’s instructions. The average sequencing depth was 262x (maximum 1321x and minimum 27x). The obtained genome coverage was not reported. Risk was rated unclear. |
| **Phelan 2016** | The ten included strains are original strains (deposited as PRJEB15555 on 2016-10-11), not mentioned to be used for training the first version of TBProfiler (Coll et al. 2015). However, since the TBProfiler version used in this study not mentioned, unclear whether these could have been added to training data. Risk was rated unclear. | Default settings (assumed) | TBProfiler: webserver. Version not mentioned  Mykrobe: version July 2016 | | Illumina sequencing according to manufacturer’s instructions. Low concern for bias. The median read depth was 53x (range: 25-141); an average of 96.4% of the genome was covered by at least 10 reads.  Low concern for bias. |
| **Phelan 2019** | Seven hundred thirteen strains from project PRJEB10385 were used for both updating the resistance mutations catalogue (Implementation section, reference (61)) and for validation (Table S4). Risk was rated high. | Default settings (expected error rate for Mykrobe 0.15 for Oxford Nanopore data, as required by default) | Mykrobe Predictor command-line version (v0.5.6-0-gbd7923a-dirty)  TBProfiler version 2.0 | | This concerns a composite dataset of public domain strains. Sequencing depth and coverage not mentioned for strains other than MinION-sequenced strains. Risk was rated unclear. |
| **Qu 2024** | These concerned original strains deposited publicly 2023-12-17. These were not included in training data of Mykrobe v.0.9.0.  Risk was rated low. | Mykrobe was used with default settings (assumed) | Mykrobe v.0.9.0 | | Illumina sequencing. An average depth of 273x was achieved. Genome coverage was not reported.  Risk was rated unclear. |
| **Quagliaro 2023** | This concerned an original dataset not mentioned to have been deposited in a public repository. Strains unlikely to have been used to train any of the evaluated tools. Risk was rated low. | Default settings (assumed) | Mykrobe: version not mentioned  PhyResSE: version not mentioned | | Illumina sequencing according to manufacturer’s instructions. Exclusion of strains with sequencing depths of below 20x. Average coverage of included strains was 98% and average depth was 119x.  Low concern for bias. |
| **Rukmana 2024** | This concerned private strains, not used in the training data of GenTB or Mykrobe.  Risk was rated low. | Mykrobe and GenTB was used with default settings (assumed) | Versions of GenTB and Mykrobe not mentioned | | Illumina sequencing by a WGS provider. Required depth and coverage not mentioned.  Risk was rated unclear. |
| **Schleusener 2017** | This dataset of 91 Sierra Leone strains was used in the initial validation study of both PhyResSE (Feuerriegel 2015), MTBseq (Kohl 2018) and ReSeqTB (Ezewudo 2018). Risk of use as training data in these tools was unclear. | Default settings (mentioned for KvarQ, assumed for the others) | KvarQ: version 0.12.1  Mykrobe Predictor version 0.1.3  PhyResSE version 1.0  TBProfiler: local tool; downloaded Jan 2016  CASTB: v.1.1 | | Illumina sequencing according to manufacturer’s instructions. Average read depth and coverage are not mentioned. Risk for bias was rated unclear. |
| **Sharma 2024** | This concerned a study of unpublished stocked isolates.  Risk was rated low. | Mykrobe and TBProfiler were used with default settings (assumed) | Mykrobe  v0.10.0  TB Profiler v4.4.1 | | Illumina sequencing at the National Microbiology Laboratory. A  minimum WGS sequence coverage depth of 30x was required. Required genome coverage not specified.  Risk was rated unclear. |
| **Silcocks 2023** | Strains were deposited as PRJNA355614 on 2017-04-19 and as PRJNA1028637 on 16-Oct-2023. It is unclear whether these strains were used in the training data of TBProfiler.  Risk was rated unclear. | TBProfiler was used with default settings (assumed) | TB-Profiler v4.2.0 | Illumina sequencing according to manufacturer’s instructions. The application of quality filtering was mentioned, but required coverage and sequencing depth were not specified. Risk was rated unclear. | |
| **Vallejos-Sanchez 2025** | These concern strains previously deposited as PRJEB5280, PRJEB32234, PRJEB23245 and PRJEB39837. Of these, PRJEB32234 PRJEB23245 were used for compiling the WHO 2021 catalogue,(44) which was also used in compiling the TBProfiler catalogue.  Risk of bias was rated high. | TBProfiler and MTBseq were used with default settings (mentioned). | TBProfiler v4.4.2  MTBseq: version not mentioned | | A composite dataset of Peruvian genomes previously made public. An average sequencing depth of 40x and genome coverage > 90% was required. Risk of bias was rated low. |
| **Van Beek 2019** | Tools were trained with other strains than those used in this novel, prospective validation set (first ENA deposition of strains 2018-05-03; ENA identification numbers in the publication’s Supplementary Table 1). Risk was rated low. | Default settings (assumed) | Mykrobe Predictor version 0.1.3 Beta  PhyResSE version 1.0  TB Profiler version 0.1  TGS-TB version 2; assessed on 9 June 2017  KvarQ: assessed 9 JUN 2017 | | Illumina sequencing according to manufacturer’s instructions. Sequencing was repeated to assure a minimum read depth of 10. The median read depth was 71 (10 to 323) (determined by PhyResSE). A separate measure for coverage was not reported.  Risk of bias was rated unclear. |
| **Wang & Dong 2025** | These concerned original strains deposited publicly 2025-06-29. It is unlikely these were included in the training data of TB-Profiler v2.8.12.  Risk was rated low. | TBProfiler was used with default settings (assumed). | TBProfiler v2.8.1 | | Illumina sequencing. Average coverage > 95% and mean depth > 100 x. Risk was rated low. |
| **Wang & Lai 2025** | These concerned original strains deposited publicly on 9-Jul-2025. It is unlikely these were included in the training data of any of the evaluated tools.  Risk was rated low. | All tools were executed with default settings (mentioned). | TBProfiler v6.2.2  PhyResSe v1.0  Mykrobe v0.10.0  SAM-TB: version not mentioned | | Illumina sequencing according to manufacturer’s instructions. An average sequencing depth of > 20 x and genome coverage > 95% was required.  Risk of bias was rated low. |
| **Wang & Yu 2025** | These concerned original strains deposited publicly 2025-06-29. It is unlikely these were included in the training data of TB-Profiler v2.8.12.  Risk was rated low. | TBProfiler was used with default settings (assumed). | TBProfiler v2.8.1 | | Illumina sequencing. Average 1x coverage 99.3%, average 10x coverage 99.2%, and mean depth 193.5x. Risk was rated low. |
| **Wu 2020** | This concerned an original dataset not mentioned to have been deposited in a public repository. Strains unlikely to have been used to train any of the evaluated tools. Risk was rated low. | Default settings (assumed) | TBProfiler: webserver in March 2020 | | Illumina sequencing according to manufacturer’s instructions. Minimum average depth was 51.67 (mean 166.29) and average genome 1x coverage 99.38. |
| **Wu 2022** | This is a prospective study using original strains, deposited as PRJNA806507 on 13-Feb-2022. Risk was rated low. | Default settings (mentioned) | TBProfiler: version not mentioned. | | Illumina sequencing performed according to manufacturer’s instructions. Genomes were excluded if they had sequencing depths of below 20x or genome coverage below 90%.  Risk was rated low. |
| **Yang 2022** | Validation strains were drawn from datasets previously used to train tools, including those described by Bradley et al. 2015 (used to train Mykrobe v.0.7.0)(1,9) and Coll et al. 2018 (PRJEB10385; used to train TBProfiler v.2.0).(3,61) Risk of bias was rated high. | Default settings (assumed) | TBProfiler: version not mentioned.  SAM-TB: first version. | | This concerns a composite dataset of public domain strains. Strains were included if the average depth was at least 50x, and a 10x coverage of at least 97% of the genome. Risk was rated low. |

# Supplementary Table S4. Extracted quality control data for Domain 3

As specified in Supplementary File S2, bias was assessed using the following signalling question: were WHO-endorsed assays for phenotypic DST used?

**Quality control data extraction table for Domain 3 (the reference test, phenotypic drug-susceptibility testing). Interpretation of bias is color coded as red (high risk of bias), grey (unclear risk of bias) or green (low risk of bias).**

|  | **Domain 3: the reference test** |
| --- | --- |
|  | **Signalling question: were WHO-endorsed assays for phenotypic DST used?** |
| **Bradley 2015** | Phenotypic DST was performed in reference laboratories with WHO-endorsed methods. Risk was rated low. |
| **Chatterjee 2017** | For 29/74 isolates pDST was performed using the MGIT 960 SIRE kit; other isolates were tested at Foundation for Medical Research, Mumbai, India; method for pDST was not mentioned for these isolates. Risk was rated unclear. |
| **Che 2022** | Phenotypic DST was performed according to WHO-recommended protocols; modalities were not specified.  Risk was rated low. |
| **Chen 2025** | Phenotypic DST modalities included both WHO endorsed and robust non-endorsed methods (UKMYC5/6 broth microdilution) in significant proportions. (44)  Risk was rated low. |
| **Cloutier Charette 2024** | Phenotypic DST was done using the proportion method on Löwenstein-Jensen medium. For an unspecified proportion of isolates, molecular tests informed the reference classification.  Risk was rated unclear. |
| **Coll 2015** | Phenotypic DST was performed by BACTEC 960 or BACTEC 460 (4/7 datasets), a solid medium method (1/7 datasets), the agar proportion method (1/7) and the ratio method (1/7). Since these concerned WHO-endorsed methods, risk was rated low. |
| **Daniyarov 2023** | Phenotypic drug-susceptibility testing was done using the BACTEC-MGIT 960 system according to manufacturer’s instructions.  Risk was rated low. |
| **Deelder 2022** | Authors mention that phenotypic DST was performed according to WHO-recommended protocols, not further specified..  Risk was rated low. |
| **Gröschel 2021** | This concerns a composite dataset with phenotypic testing with diverse protocols (Table S2). Phenotypic DST methods were unspecified for subsets of the strain set. Risk was rated unclear. |
| **Guimarães 2020** | Phenotypic DST was performed using the Proportions Method in Löwenstein-Jensen (LJ) medium according to guidelines from the national manual for laboratory surveillance of TB and other mycobacteria. Risk was rated low. |
| **Hall & Lima 2023** | This concerned a dataset of public genomes, for which phenotypic DST modalities were not specified.  Risk was rated unclear. |
| **Hall & Rabodoarivelo 2023** | Phenotypic DST was performed using the indirect proportion method on Löwenstein-Jensen medium (Malagasy isolates) and using Middlebrook 7H9 medium for the South African isolates. Phenotypic DST was not performed for British isolates.  Risk was rated low. |
| **Hasan 2023** | Strains were downloaded from ReSeqTB, a curated, quality-controlled data of mycobacterial genotype-phenotype correlated data.(19)  Risk was rated low. |
| **He 2024** | Phenotypic DST was performed according to CLSI and WHO guidelines, using the proportion method on Löwenstein-Jensen medium, and using MGIT 960 for pyrazinamide and pretonamid, using multiple testing and a consensus procedure for pyrazinamide, ethambutol and pretonamid.  Risk was rated low. |
| **Hunt 2019** | Supplementary Table S1 summarizes used pDST methods: mostly MGIT 960 (16/23 laboratories), but also MODS (1/23 laboratories), Middlebrooks 7H10 Agar (1/23), Agar proportion (1/23), Löwenstein-Jensen resistance ratio method or proportion method (4/23). Risk was rated low. |
| **Iwamoto 2019** | Phenotypic DST was performed using the MGIT AST PZA kit; the PZase assay by Wayne’s method was used for 16 strains that showed discrepant results. MIC results were determined for 7 out of 16 strains with persistent discrepant results using the MGIT EpiCenter system. Risk was rated low. |
| **Johnsen 2019** | This concerned a dataset of public genomes, for which phenotypic DST modalities were not specified.  Risk was rated unclear. |
| **Kardan-Yamchi 2020** | Phenotypic DST was performed using the proportion method on Löwenstein-Jensen medium by an accredited laboratory. Risk was rated low. |
| **Lim 2023** | Phenotypic testing done using the MGIT system in Singapore’s reference laboratory.  Risk was rated low. |
| **Liu 2024** | Phenotypic testing was done using the agar proportion method, except for pyrazinamide using the Bactec MGIT 960 system, according to WHO-recommendations.  Risk was rated low. |
| **Mahé 2019** | All strains came from datasets that were curated and/or that reported results obtained in laboratories part of the WHO supranational laboratory network (Coll 2015). Risk was rated low. |
| **Morey-Léon 2023** | Phenotypic DST was performed using the Bactec MGIT 960 System in 67 samples and using an agar proportion assay for 21 samples, according to WHO recommendations.  Risk was rated low. |
| **Peker 2021** | Modalities for phenotypic drug-susceptibility testing were not reported.  Risk was rated unclear. |
| **Phelan 2016** | Phenotypic DST was performed using the BACTEC 960 according to manufacturer’s instructions. Risk was rated low. |
| **Phelan 2019** | This concerns a composite dataset without documentation of the phenotypic reference test used. Risk was rated unclear. |
| **Qu 2024** | The TREK Sensititre MYCOTB plates were used for phenotypic DST, a commercial method currently unendorsed by WHO.  Risk was rated low. |
| **Quagliaro 2023** | Phenotypic DST was performed using the BACTEC 960 according to manufacturer’s instructions. Risk was rated low. |
| **Rukmana 2024** | Phenotypic DST was done using the BACTEC MGIT960 PZA Kit in a certificed reference laboratory.  Risk was rated low. |
| **Schleusener 2017** | Phenotypic DST performed using the proportion method on Löwenstein-Jensen medium, the modified proportion method in the BACTEC 460 or BACTEC 960 system. Risk was rated low. |
| **Sharma 2024** | Phenotypic DST was performed using the BACTEC 960 system according to manufacturer’s recommendations.  Risk was rated low. |
| **Silcocks 2023** | Phenotypic DST was performed using the BACTEC MGIT 960 SIRE kit according to the manufacturer’s instructions.  Risk was rated low. |
| **Vallejos-Sanchez 2025** | A WHO-endorsed microscopic observation drug susceptibility (MODS) assay was used.  Risk was rated low. |
| **Van Beek 2019** | Phenotypic DST was performed using the MGIT system according to manufacturer’s instructions. Risk was rated low. |
| **Wang & Dong 2025** | Phenotypic DST was done using a custom broth microdilution plate (Baso, Zhuhai, China). Risk was rated unclear. |
| **Wang & Lai 2025** | The indirect proportion method on Löwenstein-Jenssen solid medium was used, according to WHO recommendations. Risk was rated low. |
| **Wang & Yu 2025** | Phenotypic DST was done by an accredited laboratory using the proportion method. Risk was rated low. |
| **Wu 2020** | Phenotypic DST was performed using the Bactec 960 system according to manufacturer’s instructions. Risk was rated low. |
| **Wu 2022** | Phenotypic DST using the MycoTB system (Trek Diagnostic Systems, Thermo Fisher Scientific, USA), a non-WHO-endorsed method.  Risk was rated unclear. |
| **Yang 2022** | This concerns a composite dataset without documentation of the phenotypic reference test used. Risk was rated unclear. |

# Supplementary Table S5. Systematic review of genomic prediction tools

**Supplementary Table S5.** Characteristics of public genomic prediction tools. Columns shaded red correspond to tools for which no public updates have been released in five years before search date of this systematic review, or that have been discontinued by their authors.

|  | **KvarQ** | **TGS-TB** | **CASTB** | **PhyResSE** | **ReSeqTB-UVP** | **MTBseq** | **TBProfiler** | **Mykrobe** | **GenTB** | **ResFinder-PointFinder** | **SAM-TB** |
| --- | --- | --- | --- | --- | --- | --- | --- | --- | --- | --- | --- |
| **Year of last public release*** | 2014 | 2015 | 2017 | 2021 | 2021 | 2023 | 2025 | 2024 | 2022 | 2023 | 2026 |
| **Format** | GUI and command-line utlility | Webserver | Webserver | Webserver | Webserver and command-line | Command-line | Webserver and command-line | GUI and command-line | Webserver and command-line | Webserver and command-line | Webserver |
| **Open-source code** | Yes | Yes | No | Yes | Yes | Yes | Yes | Yes | Yes | Yes | No |
| **Preprocessing** | Read trimming, phred score user-supplied | Read trimming using Skewer, phred threshold 15 | Read contamination check (BIGSdb) | FastQValidator, contamination check, FastQC | FastQValidator, Kraken contamination check, FastQC, Prinseq | No | Read trimming using Trimmomatic | No | FastQValidator, read trimming with fastp, Kraken contamination check, read filtering seqtk | KMA (K-mer Alignment) handles raw read mapping/quality internally. | Read trimming for Phred quality cutoff 20 and read length > 35. |
| **Genome assembly tools (reference genome)** | No genome assembly | BWA-MEM (H37Rv) | A velvet *de novo* assembler (not specified) | BWA-MEM (H37Rv), duplicate read removal (SAMtools), Qualimap, base recalibration and indel realignment (GATK) | BWA-MEM (H37Rv), duplicate read removal (PICARD), base recalibration and indel realignment (GATK) | BWA-MEM (H37Rv or user-supplied), duplicate read removal (SAMtools), base recalibration and indel realignment (GATK) | Assembly using BWA, Bowtie or Minimap2 (H37Rv or user-supplied) | De Bruijn graph construction (custom) | Paired-read matching (fastq-pair), minimap2 alignment (H37Rv), duplicate read removal (SAMtools) | Gene-based alignment: uses KMA for reads or BLASTn for assemblies. Reference genes from H37Rv. | Assembly using BWA-MEM (H37Rv). SAMtools for quality control. Read deduplication using Picard. |
| **Variant calling tools** | Reads mapped to hypothetical ancestral MTBC genome; parameters minreadlength, minoverlap, maxerrors are customizable | KvarQ used for DST; variant-calling for phylogenetic analysis if ≥ 5x coverage and phred score ≥ 20; cutoffs not customizable | Using MUMmer; coverage, frequency and phred score thresholds not specified; thresholds not customizable | Using GATK; coverage, frequency, phred score thresholds not specified and not customizable | Using GATK and SAMtools; base call and mapping quality phred score ≥ 20; coverage ≥ 10x (in addition to other criteria) | Using SAMtools; coverage ≥ 4 forward and reverse reads, frequency 75%, ≥ 4 calls with phred score ≥ 20; user customizable | Using Freebayes, BCFtools or gatk and Delly; Default thresholds are depth ≥ 10 and allele frequency ≥   10%; both customizable | Custom algorithm based on De Bruijn graph comparison. Required variant depth can be customized | Using Pilon with default parameters and frequency threshold 40%; not customizable | Direct sequence comparison (KMA/BLAST). Thresholds: >60% gene coverage, >90% identity. | SAMtools/VarScan used for SNP calling and short indels. Delly used for large deletions. Variants must have depth ≥ 5 and frequency of ≤ 75% (+ other criteria) |
| **Annotation algorithm** | Direct association, binary | Direct association, binary | Direct association, binary | Direct association, binary | Direct association (SnpEff), confidence-graded | Direct association; binary | Direct association, binary | Direct association, binary | Random forest classifier, wide-and-deep neural network | Direct association. Includes loss-of-function logic (e.g. truncations) | Direct association, binary |
| **Species identification** | No | Unclear | MTBC, NTM | MTBC | MTBC | MTBC | MTBC | MTBC, NTM | No | No | MTB, NTM |
| **Lineage classification** | No | Yes | Yes | Yes | Yes | Yes | Yes | Yes | Yes | No | Yes |
| **Strain typing** | No | IS6110, RFLP,  Spoligotyping,  MIRU-VNTR,  SNP-based | Spoligotyping  MIRU-VNTR  SNP concatemer | No | No | SNP distance matrix | Spoligotype, SNP distance matrix, exports Newick files for phylogenetic tree reconstruction | No | No | No | SNP distance matrix and phylogenetic tree reconstruction |
| **Plain language report** | No | Yes | Yes | Yes | Yes | Yes | Yes | Yes | Yes | Yes | Yes |

NTM, nontuberculous mycobacteria; MTBC, *Mycobacterium tuberculosis* complex; DST, drug-susceptibility testing; GUI, graphical user-interface.

* Year of last public release as found on the Github page of the respective tool (for KvarQ, MTBseq, TBProfiler, Mykrobe, ResFinder/PointFinder, GenTB) or as found on the developer’s webpage for CASTB (<http://castb.ri.ncgm.go.jp/CASTB/#History>, accessed 29 Oct 2022). PhyResSE’s genetic variation catalogue was last updated using the WHO catalogue of resistance mutations (64) in 2021 (contact author Dr. Viola Dreyer in February 2026). The TGS-TB website (<https://gph.niid.go.jp/>) has been unavailable since August 2020 at the latest (https://web.archive.org/web/20200804142300/https://gph.niid.go.jp/). No other sources attest to maintenance of the tool since its release in 2015.

# Supplementary Figure S1: Diagnostic performance of whole-genome sequencing (WGS) tools for miscellaneous group A, B and C drugs.

**Supplementary Figure S1. Diagnostic performance of whole-genome sequencing (WGS) tools for miscellaneous group A, B and C drugs**. Pooled estimates of sensitivity (top panels) and specificity (bottom panels) are shown for each tool across different drugs. Points represent the pooled estimate with 95% confidence intervals. Dotted horizontal lines indicate the 2021 WHO target product profile minimum thresholds where available: ≥80% sensitivity/≥98% specificity for bedaquiline, linezolid, clofazimine and delamanid; no target for the other agents has been specified.(65)  Dashes denote estimates calculated by pooling raw counts (due to insufficient studies <3 for meta-analysis); these values should be interpreted with caution as they do not account for between-study heterogeneity. Note the missing sensitivity markers for clofazimine (CLO), bedaquiline (BDQ), and linezolid (LIN), reflecting the absence of phenotypically resistant isolates in the analyzed study cohorts. ETH, ethionamide; PRO, prothionamide; PAS, para-aminosalicylic acid; CYC, cycloserine; CLO, clofazimine; BDQ, bedaquiline; DEL, delamanid; LIN, linezolid.

# Supplementary Figure S2. Meta-regression analysis of continuous covariates on diagnostic performance for fluoroquinolones and second-line injectables.

**Supplementary Figure S2. Meta-regression analysis of continuous covariates on diagnostic performance for fluoroquinolones and second-line injectables.** Forest plots showing the effect (ß on logit scale) of lineage proportions, RIF resistance prevalence, publication year, and study size on sensitivity (circles) and specificity (triangles). Filled blue markers indicate significant effects after FDR correction (q < 0.05); filled gray markers indicate nominal significance (p < 0.05); hollow markers indicate non-significant results. Red asterisks (*) denote estimates based on limited data (N < 10 studies) and should be interpreted with caution. Arrows indicate effect sizes exceeding the plot range.

# Supplementary Figure S3. Meta-regression analysis of categorical covariates on diagnostic performance for fluoroquinolones and second-line injectables

**Supplementary Figure S3. Meta-regression analysis of categorical covariates on diagnostic performance for fluoroquinolones and second-line injectables.** Forest plots comparing effect sizes (ß on logit scale) for phenotype testing method (reference: solid), data source (reference: original), and geographic region (reference: high-income [HIC] or western countries). Circles represent sensitivity effects; triangles represent specificity. Filled blue markers indicate significant differences relative to the reference group (FDR q < 0.05); filled gray markers indicate nominal significance (p < 0.05); hollow markers are non-significant. Effects > 0 indicate higher sensitivity/specificity compared to the reference. (L)MIC includes both Lower-Middle Income Countries and Upper-Middle Income Countries.

# References

1.	Hunt M, Bradley P, Lapierre SG, Heys S, Thomsit M, Hall MB, et al. Antibiotic resistance prediction for Mycobacterium tuberculosis from genome sequence data with Mykrobe. Wellcome Open Res. 2019 Dec 2;4:191. doi:10.12688/wellcomeopenres.15603.1 PubMed PMID: 32055708; PubMed Central PMCID: PMC7004237.

2.	Phelan J, O’Sullivan DM, Machado D, Ramos J, Whale AS, O’Grady J, et al. The variability and reproducibility of whole genome sequencing technology for detecting resistance to anti-tuberculous drugs. Genome medicine. 2016;8(1):1.

3.	Phelan JE, O’Sullivan DM, Machado D, Ramos J, Oppong YEA, Campino S, et al. Integrating informatics tools and portable sequencing technology for rapid detection of resistance to anti-tuberculous drugs. Genome Medicine. 2019 Jun 24;11(1):41. doi:10.1186/s13073-019-0650-x

4.	Mahé P, El Azami M, Barlas P, Tournoud M. A large scale evaluation of TBProfiler and Mykrobe for antibiotic resistance prediction in Mycobacterium tuberculosis. PeerJ. 2019;7:e6857.

5.	Schleusener V, Köser CU, Beckert P, Niemann S, Feuerriegel S. Mycobacterium tuberculosis resistance prediction and lineage classification from genome sequencing: comparison of automated analysis tools. Scientific reports. 2017;7:46327.

6.	van Beek J, Haanperä M, Smit PW, Mentula S, Soini H. Evaluation of whole genome sequencing and software tools for drug susceptibility testing of Mycobacterium tuberculosis. Clinical Microbiology and Infection. 2019;25(1):1.

7.	Gröschel MI, Owens M, Freschi L, Vargas R, Marin MG, Phelan J, et al. GenTB: A user-friendly genome-based predictor for tuberculosis resistance powered by machine learning [Internet]. 2021 Mar [cited 2021 Sep 27]. p. 2021.03.27.437319. Available from: https://www.biorxiv.org/content/10.1101/2021.03.27.437319v1 doi:10.1101/2021.03.27.437319

8.	Ngo TM, Teo YY. Genomic prediction of tuberculosis drug-resistance: benchmarking existing databases and prediction algorithms. BMC bioinformatics. 2019;20(1):1.

9.	Bradley P, Gordon NC, Walker TM, Dunn L, Heys S, Huang B, et al. Rapid antibiotic-resistance predictions from genome sequence data for Staphylococcus aureus and Mycobacterium tuberculosis. Nature communications. 2015;6(1):1.

10.	Coll F, McNerney R, Preston MD, Guerra-Assunção JA, Warry A, Hill-Cawthorne G, et al. Rapid determination of anti-tuberculosis drug resistance from whole-genome sequences. Genome Med. 2015;7(1):1. doi:10.1186/s13073-015-0164-0 PubMed PMID: 26019726; PubMed Central PMCID: PMC4446134.

11.	Kohl TA, Utpatel C, Schleusener V, De Filippo MR, Beckert P, Cirillo DM, et al. MTBseq: a comprehensive pipeline for whole genome sequence analysis of Mycobacterium tuberculosis complex isolates. PeerJ. 2018;6:e5895.

12.	Feuerriegel S, Schleusener V, Beckert P, Kohl TA, Miotto P, Cirillo DM, et al. PhyResSE: a Web Tool Delineating Mycobacterium tuberculosis Antibiotic Resistance and Lineage from Whole-Genome Sequencing Data. J Clin Microbiol. 2015 Jun;53(6):6. doi:10.1128/JCM.00025-15 PubMed PMID: 25854485; PubMed Central PMCID: PMC4432036.

13.	Heupink TH, Verboven L, Warren RM, Van Rie A. Comprehensive and accurate genetic variant identification from contaminated and low-coverage Mycobacterium tuberculosis whole genome sequencing data. Microb Genom. 2021 Nov;7(11):000689. doi:10.1099/mgen.0.000689 PubMed PMID: 34793294; PubMed Central PMCID: PMC8743552.

14.	Dreyer V, Utpatel C, Kohl TA, Barilar I, Gröschel MI, Feuerriegel S, et al. Detection of low-frequency resistance-mediating SNPs in next-generation sequencing data of Mycobacterium tuberculosis complex strains with binoSNP. Sci Rep. 2020 May 12;10(1):7874. doi:10.1038/s41598-020-64708-8 PubMed PMID: 32398743; PubMed Central PMCID: PMC7217866.

15.	Green AG, Yoon CH, Chen ML, Ektefaie Y, Fina M, Freschi L, et al. A convolutional neural network highlights mutations relevant to antimicrobial resistance in Mycobacterium tuberculosis. Nat Commun. 2022 Jul 2;13(1):3817. doi:10.1038/s41467-022-31236-0 PubMed PMID: 35780211; PubMed Central PMCID: PMC9250494.

16.	Ramos-García AA, Mejía-Ponce PM, Sélem-Mojica N, Santos-Díaz A, Martínez-Ledesma E, Licona-Cassani C. FuNTB: a functional network clustering tool for the analysis of genome-wide genetic variants in Mycobacterium tuberculosis. Bioinformatics. 2025 Jul 1;41(7):btaf341. doi:10.1093/bioinformatics/btaf341 PubMed PMID: 40498551; PubMed Central PMCID: PMC12255883.

17.	Drouin A, Letarte G, Raymond F, Marchand M, Corbeil J, Laviolette F. Interpretable genotype-to-phenotype classifiers with performance guarantees. Sci Rep. 2019 Mar 11;9:4071. doi:10.1038/s41598-019-40561-2 PubMed PMID: 30858411; PubMed Central PMCID: PMC6411721.

18.	Steiner A, Stucki D, Coscolla M, Borrell S, Gagneux S. KvarQ: targeted and direct variant calling from fastq reads of bacterial genomes. BMC Genomics. 2014 Oct 9;15(1):1. doi:10.1186/1471-2164-15-881

19.	Ezewudo M, Borens A, Chiner-Oms Á, Miotto P, Chindelevitch L, Starks AM, et al. Integrating standardized whole genome sequence analysis with a global Mycobacterium tuberculosis antibiotic resistance knowledgebase. Sci Rep. 2018 Oct 18;8(1):15382. doi:10.1038/s41598-018-33731-1

20.	Yang T, Gan M, Liu Q, Liang W, Tang Q, Luo G, et al. SAM-TB: a whole genome sequencing data analysis website for detection of Mycobacterium tuberculosis drug resistance and transmission. Briefings in Bioinformatics. 2022 Mar 1;23(2):2. doi:10.1093/bib/bbac030

21.	Iwai H, Kato-Miyazawa M, Kirikae T, Miyoshi-Akiyama T. CASTB (the comprehensive analysis server for the Mycobacterium tuberculosis complex): A publicly accessible web server for epidemiological analyses, drug-resistance prediction and phylogenetic comparison of clinical isolates. Tuberculosis. 2015 Dec 1;95(6):6. doi:10.1016/j.tube.2015.09.002

22.	Heupink TH, Verboven L, Sharma A, Rennie V, de Diego Fuertes M, Warren RM, et al. The MAGMA pipeline for comprehensive genomic analyses of clinical Mycobacterium tuberculosis samples. PLoS Comput Biol. 2023 Nov;19(11):e1011648. doi:10.1371/journal.pcbi.1011648 PubMed PMID: 38019772; PubMed Central PMCID: PMC10686480.

23.	Sekizuka T, Yamashita A, Murase Y, Iwamoto T, Mitarai S, Kato S, et al. TGS-TB: Total Genotyping Solution for Mycobacterium tuberculosis Using Short-Read Whole-Genome Sequencing. PLOS ONE. 2015 Nov 13;10(11):11. doi:10.1371/journal.pone.0142951

24.	Johnsen CH, Clausen PTLC, Aarestrup FM, Lund O. Improved Resistance Prediction in Mycobacterium tuberculosis by Better Handling of Insertions and Deletions, Premature Stop Codons, and Filtering of Non-informative Sites. Front Microbiol. 2019;10:2464. doi:10.3389/fmicb.2019.02464 PubMed PMID: 31736907; PubMed Central PMCID: PMC6834686.

25.	Olawoye IB, Frost SDW, Happi CT. The Bacteria Genome Pipeline (BAGEP): an automated, scalable workflow for bacteria genomes with Snakemake. PeerJ. 2020;8:e10121. doi:10.7717/peerj.10121 PubMed PMID: 33194387; PubMed Central PMCID: PMC7597632.

26.	Muzondiwa D, Mutshembele A, Pierneef RE, Reva ON. Resistance Sniffer: An online tool for prediction of drug resistance patterns of Mycobacterium tuberculosis isolates using next generation sequencing data. International Journal of Medical Microbiology. 2020 Feb 1;310(2):2. doi:10.1016/j.ijmm.2020.151399

27.	Bogaerts B, Delcourt T, Soetaert K, Boarbi S, Ceyssens PJ, Winand R, et al. A Bioinformatics Whole-Genome Sequencing Workflow for Clinical Mycobacterium tuberculosis Complex Isolate Analysis, Validated Using a Reference Collection Extensively Characterized with Conventional Methods and In Silico Approaches. J Clin Microbiol. 2021 May 19;59(6):e00202-21. doi:10.1128/JCM.00202-21 PubMed PMID: 33789960; PubMed Central PMCID: PMC8316078.

28.	van Heusden P, Mashologu Z, Lose T, Warren R, Christoffels A. The COMBAT-TB Workbench: Making Powerful Mycobacterium tuberculosis Bioinformatics Accessible. mSphere. 2022 Feb 23;7(1):e0099121. doi:10.1128/msphere.00991-21 PubMed PMID: 35138128; PubMed Central PMCID: PMC8827006.

29.	Swargam S, Kumari I, Kumar A, Pradhan D, Alam A, Singh H, et al. MycoVarP: Mycobacterium Variant and Drug Resistance Prediction Pipeline for Whole-Genome Sequence Data Analysis. Front Bioinform. 2022 Jun 3;1. doi:10.3389/fbinf.2021.805338

30.	Hall MB, Lima L, Coin LJM, Iqbal Z. Drug resistance prediction for Mycobacterium tuberculosis with reference graphs. Microb Genom. 2023 Aug;9(8):mgen001081. doi:10.1099/mgen.0.001081 PubMed PMID: 37552534; PubMed Central PMCID: PMC10483414.

31.	Singhal R, Hingane S, Bhalla M, Sharma A, Ferdosh S, Tiwari A, et al. Evaluation of AAICare®-TB sequence analysis tool for accurate diagnosis of drug-resistant tuberculosis: A comparative study with TB-Profiler and Mykrobe. Tuberculosis (Edinb). 2024 Jul;147:102515. doi:10.1016/j.tube.2024.102515 PubMed PMID: 38744006.

32.	Kim D, Shin JI, Yoo IY, Jo S, Chu J, Cho WY, et al. GenoMycAnalyzer: a web-based tool for species and drug resistance prediction for Mycobacterium genomes. BMC Genomics. 2024 Apr 20;25:387. doi:10.1186/s12864-024-10320-3 PubMed PMID: 38643090; PubMed Central PMCID: PMC11031912.

33.	Wang Y, Jiang Z, Liang P, Liu Z, Cai H, Sun Q. TB-DROP: deep learning-based drug resistance prediction of Mycobacterium tuberculosis utilizing whole genome mutations. BMC Genomics. 2024 Feb 12;25(1):167. doi:10.1186/s12864-024-10066-y PubMed PMID: 38347478; PubMed Central PMCID: PMC10860279.

34.	Pal A, Mohanty D. Machine learning-based approach for identification of new resistance associated mutations from whole genome sequences of Mycobacterium tuberculosis. Bioinform Adv. 2025;5(1):vbaf050. doi:10.1093/bioadv/vbaf050 PubMed PMID: 40125545; PubMed Central PMCID: PMC11930343.

35.	Whiting PF, Rutjes AWS, Westwood ME, Mallett S, Deeks JJ, Reitsma JB, et al. QUADAS-2: a revised tool for the quality assessment of diagnostic accuracy studies. Ann Intern Med. 2011 Oct 18;155(8):8. doi:10.7326/0003-4819-155-8-201110180-00009 PubMed PMID: 22007046.

36.	Papaventsis D, Casali N, Kontsevaya I, Drobniewski F, Cirillo DM, Nikolayevskyy V. Whole genome sequencing of Mycobacterium tuberculosis for detection of drug resistance: a systematic review. Clin Microbiol Infect. 2017 Feb;23(2):2. doi:10.1016/j.cmi.2016.09.008 PubMed PMID: 27665704.

37.	Heyckendorf J, Andres S, Köser CU, Olaru ID, Schön T, Sturegård E, et al. What Is Resistance? Impact of Phenotypic versus Molecular Drug Resistance Testing on Therapy for Multi- and Extensively Drug-Resistant Tuberculosis. Antimicrob Agents Chemother. 2018 Jan 25;62(2):2. doi:10.1128/AAC.01550-17 PubMed PMID: 29133554; PubMed Central PMCID: PMC5786814.

38.	Kardan-Yamchi J, Kazemian H, Battaglia S, Abtahi H, Foroushani AR, Hamzelou G, et al. Whole Genome Sequencing Results Associated with Minimum Inhibitory Concentrations of 14 Anti-Tuberculosis Drugs among Rifampicin-Resistant Isolates of Mycobacterium Tuberculosis from Iran. J Clin Med. 2020 Feb 7;9(2):2. doi:10.3390/jcm9020465 PubMed PMID: 32046149; PubMed Central PMCID: PMC7073636.

39.	Hall MB, Rabodoarivelo MS, Koch A, Dippenaar A, George S, Grobbelaar M, et al. Evaluation of Nanopore sequencing for Mycobacterium tuberculosis drug susceptibility testing and outbreak investigation: a genomic analysis. The Lancet Microbe. 2023 Feb 1;4(2):2. doi:10.1016/S2666-5247(22)00301-9 PubMed PMID: 36549315.

40.	Bainomugisa A, Duarte T, Lavu E, Pandey S, Coulter C, Marais BJ, et al. A complete high-quality MinION nanopore assembly of an extensively drug-resistant Mycobacterium tuberculosis Beijing lineage strain identifies novel variation in repetitive PE/PPE gene regions. Microb Genom. 2018 Jul;4(7):7. doi:10.1099/mgen.0.000188 PubMed PMID: 29906261; PubMed Central PMCID: PMC6113869.

41.	Horne DJ, Pinto LM, Arentz M, Lin SYG, Desmond E, Flores LL, et al. Diagnostic Accuracy and Reproducibility of WHO-Endorsed Phenotypic Drug Susceptibility Testing Methods for First-Line and Second-Line Antituberculosis Drugs. Journal of Clinical Microbiology. 2013 Feb 1;51(2):2. doi:10.1128/JCM.02724-12

42.	Van Deun A, Aung KJM, Bola V, Lebeke R, Hossain MA, de Rijk WB, et al. Rifampin Drug Resistance Tests for Tuberculosis: Challenging the Gold Standard. Journal of Clinical Microbiology. 2013 Aug;51(8):2633–40. doi:10.1128/jcm.00553-13

43.	Chang KC, Yew WW, Zhang Y. Pyrazinamide susceptibility testing in Mycobacterium tuberculosis: a systematic review with meta-analyses. Antimicrob Agents Chemother. 2011 Oct;55(10):4499–505. doi:10.1128/AAC.00630-11 PubMed PMID: 21768515; PubMed Central PMCID: PMC3186960.

44.	Walker TM, Miotto P, Köser CU, Fowler PW, Knaggs J, Iqbal Z, et al. The 2021 WHO catalogue of Mycobacterium tuberculosis complex mutations associated with drug resistance: a genotypic analysis. Lancet Microbe. 2022 Apr;3(4):e265–73. doi:10.1016/S2666-5247(21)00301-3 PubMed PMID: 35373160; PubMed Central PMCID: PMC7612554.

45.	Hall L, Jude KP, Clark SL, Dionne K, Merson R, Boyer A, et al. Evaluation of the Sensititre MycoTB plate for susceptibility testing of the Mycobacterium tuberculosis complex against first- and second-line agents. J Clin Microbiol. 2012 Nov;50(11):3732–4. doi:10.1128/JCM.02048-12 PubMed PMID: 22895034; PubMed Central PMCID: PMC3486270.

46.	The Internet Archive. https://gph.niid.go.jp/ on the Internet Archive Wayback Machine [Internet]. 2022 [cited 2022 May 2]. Available from: https://web.archive.org/web/*/https://gph.niid.go.jp/

47.	The Internet Archive. https://platform.reseqtb.org/ on the Internet Archive Wayback Machine [Internet]. 2021 [cited 2022 Apr 29]. Available from: https://web.archive.org/web/20210410223555/https://platform.reseqtb.org/

48.	Zankari E, Allesøe R, Joensen KG, Cavaco LM, Lund O, Aarestrup FM. PointFinder: a novel web tool for WGS-based detection of antimicrobial resistance associated with chromosomal point mutations in bacterial pathogens. J Antimicrob Chemother. 2017 Oct 1;72(10):2764–8. doi:10.1093/jac/dkx217 PubMed PMID: 29091202; PubMed Central PMCID: PMC5890747.

49.	Daniyarov A, Akhmetova A, Rakhimova S, Abilova Z, Yerezhepov D, Chingissova L, et al. Whole-Genome Sequence-Based Characterization of Pre-XDR M. tuberculosis Clinical Isolates Collected in Kazakhstan. Diagnostics (Basel). 2023 Jun 8;13(12). doi:10.3390/diagnostics13122005 PubMed PMID: 37370900; PubMed Central PMCID: PMC10296843.

50.	Cohen KA, Manson AL, Desjardins CA, Abeel T, Earl AM. Deciphering drug resistance in Mycobacterium tuberculosis using whole-genome sequencing: progress, promise, and challenges. Genome Medicine. 2019 Jul 25;11(1):1. doi:10.1186/s13073-019-0660-8

51.	CRyPTIC Consortium and the 100,000 Genomes Project, Allix-Béguec C, Arandjelovic I, Bi L, Beckert P, Bonnet M, et al. Prediction of Susceptibility to First-Line Tuberculosis Drugs by DNA Sequencing. N Engl J Med. 2018 Oct 11;379(15):15. doi:10.1056/NEJMoa1800474 PubMed PMID: 30280646; PubMed Central PMCID: PMC6121966.

52.	Deelder W, Christakoudi S, Phelan J, Benavente ED, Campino S, McNerney R, et al. Machine Learning Predicts Accurately Mycobacterium tuberculosis Drug Resistance From Whole Genome Sequencing Data. Front Genet. 2019;10:922. doi:10.3389/fgene.2019.00922 PubMed PMID: 31616478; PubMed Central PMCID: PMC6775242.

53.	Yang Y, Walker TM, Walker AS, Wilson DJ, Peto TEA, Crook DW, et al. DeepAMR for predicting co-occurrent resistance of Mycobacterium tuberculosis. Bioinformatics. 2019 Sep 15;35(18):18. doi:10.1093/bioinformatics/btz067

54.	Kouchaki S, Yang Y, Walker TM, Sarah Walker A, Wilson DJ, Peto TEA, et al. Application of machine learning techniques to tuberculosis drug resistance analysis. Bioinformatics. 2019 Jul 1;35(13):13. doi:10.1093/bioinformatics/bty949 PubMed PMID: 30462147; PubMed Central PMCID: PMC6596891.

55.	Meehan CJ, Goig GA, Kohl TA, Verboven L, Dippenaar A, Ezewudo M, et al. Whole genome sequencing of Mycobacterium tuberculosis: current standards and open issues. Nat Rev Microbiol. 2019 Sep;17(9):9. doi:10.1038/s41579-019-0214-5

56.	Wyres KL, Conway TC, Garg S, Queiroz C, Reumann M, Holt K, et al. WGS Analysis and Interpretation in Clinical and Public Health Microbiology Laboratories: What Are the Requirements and How Do Existing Tools Compare? Pathogens. 2014 Jun;3(2):2. doi:10.3390/pathogens3020437

57.	Walker TM, Kohl TA, Omar SV, Hedge J, Del Ojo Elias C, Bradley P, et al. Whole-genome sequencing for prediction of Mycobacterium tuberculosis drug susceptibility and resistance: a retrospective cohort study. Lancet Infect Dis. 2015 Oct;15(10):10. doi:10.1016/S1473-3099(15)00062-6 PubMed PMID: 26116186; PubMed Central PMCID: PMC4579482.

58.	Conceição EC, Guimarães AE dos S, Lopes ML, Furlaneto IP, Rodrigues YC, da Conceição ML, et al. Analysis of potential household transmission events of tuberculosis in the city of Belem, Brazil. Tuberculosis. 2018 Dec 1;113:125–9. doi:10.1016/j.tube.2018.09.011

59.	Chen X, He G, Wang S, Lin S, Chen J, Zhang W. Evaluation of Whole-Genome Sequence Method to Diagnose Resistance of 13 Anti-tuberculosis Drugs and Characterize Resistance Genes in Clinical Multi-Drug Resistance Mycobacterium tuberculosis Isolates From China. Front Microbiol. 2019;10:1741. doi:10.3389/fmicb.2019.01741 PubMed PMID: 31417530; PubMed Central PMCID: PMC6685394.

60.	Chen X, He G, Wang S, Lin S, Chen J, Zhang W. Evaluation of Whole-Genome Sequence Method to Diagnose Resistance of 13 Anti-tuberculosis Drugs and Characterize Resistance Genes in Clinical Multi-Drug Resistance Mycobacterium tuberculosis Isolates From China. Front Microbiol. 2019 Jul 31;10:1741. doi:10.3389/fmicb.2019.01741 PubMed PMID: 31417530; PubMed Central PMCID: PMC6685394.

61.	Coll F, Phelan J, Hill-Cawthorne GA, Nair MB, Mallard K, Ali S, et al. Genome-wide analysis of multi- and extensively drug-resistant Mycobacterium tuberculosis. Nat Genet. 2018 Feb;50(2):307–16. doi:10.1038/s41588-017-0029-0

62.	Homolka S, Post E, Oberhauser B, George AG, Westman L, Dafae F, et al. High genetic diversity among Mycobacterium tuberculosis complex strains from Sierra Leone. BMC Microbiology. 2008 Jun 25;8(1):1. doi:10.1186/1471-2180-8-103

63.	GaryNapier. GaryNapier/tb-lineages [Python] [Internet]. 2023 [cited 2025 Feb 21]. Available from: https://github.com/GaryNapier/tb-lineages

64.	Walker TM, Miotto P, Köser CU, Fowler PW, Knaggs J, Iqbal Z, et al. The 2021 WHO catalogue of Mycobacterium tuberculosis complex mutations associated with drug resistance: a genotypic analysis. The Lancet Microbe. 2022 Apr 1;3(4):4. doi:10.1016/S2666-5247(21)00301-3

65.	World Health Organization. Target product profile for next-generation drug-susceptibility testing at peripheral centres [Internet]. 2021 [cited 2023 Feb 13]. Available from: https://www.who.int/publications-detail-redirect/9789240032361
